# The CHEMDNER corpus of chemicals and drugs and its annotation principles

**DOI:** 10.1186/1758-2946-7-S1-S2

**Published:** 2015-01-19

**Authors:** Martin Krallinger, Obdulia Rabal, Florian Leitner, Miguel Vazquez, David Salgado, Zhiyong Lu, Robert Leaman, Yanan Lu, Donghong Ji, Daniel M Lowe, Roger A Sayle, Riza Theresa Batista-Navarro, Rafal Rak, Torsten Huber, Tim Rocktäschel, Sérgio Matos, David Campos, Buzhou Tang, Hua Xu, Tsendsuren Munkhdalai, Keun Ho Ryu, SV Ramanan, Senthil Nathan, Slavko Žitnik, Marko Bajec, Lutz Weber, Matthias Irmer, Saber A Akhondi, Jan A Kors, Shuo Xu, Xin An, Utpal Kumar Sikdar, Asif Ekbal, Masaharu Yoshioka, Thaer M Dieb, Miji Choi, Karin Verspoor, Madian Khabsa, C Lee Giles, Hongfang Liu, Komandur Elayavilli Ravikumar, Andre Lamurias, Francisco M Couto, Hong-Jie Dai, Richard Tzong-Han Tsai, Caglar Ata, Tolga Can, Anabel Usié, Rui Alves, Isabel Segura-Bedmar, Paloma Martínez, Julen Oyarzabal, Alfonso Valencia

**Affiliations:** 1 Structural Computational Biology Group, Structural Biology and BioComputing Programme, Spanish National Cancer Research Centre, Madrid, Spain; 2 Small Molecule Discovery Platform, Molecular Therapeutics Program, Center for Applied Medical Research (CIMA), University of Navarra, Pamplona, Spain; 3 Computational Intelligence Group, Department of Artificial Intelligence, Universidad Politecnica de Madrid, Madrid, Spain; 4 Faculte de Medecine La Timone, Marseille, Marseille, France; 5 National Center for Biotechnology Information (NCBI), National Institutes of Health, Bethesda, USA; 6 Natural Language Processing Lab, Wuhan University, Wuhan, Hubei, PR China; 7 NextMove Software Ltd, Innovation Centre, Unit 23, Science Park, Milton Road, Cambridge, UK; 8 National Centre for Text Mining, Manchester Institute of Biotechnology, Manchester, UK; 9 Humboldt-Universität zu Berlin, Knowledge Management in Bioinformatics, Berlin, Germany; 10 Department of Computer Science, University College London, London, UK; 11 IEETA/DETI, University of Aveiro, Campus Universitario de Santiago, Aveiro, Portugal; 12 Department of Computer Science, Harbin Institute of Technology, Shenzhen Graduate School Shenzhen, GuangDong, PR China; 13 School of Biomedical Informatics, The University of Texas Health Science Center at Houston, Houston, USA; 14 Database/Bioinformatics Laboratory, School of Electrical and Computer Engineering, Chungbuk National University, Cheongju, South Korea; 15 RelAgent Pvt Ltd, IIT Madras Research Park, Taramani, Chennai, India; 16 Faculty of computer and information science, University of Ljubljana, Ljubljana, Slovenia; 17 OntoChem GmbH, Halle, Germany; 18 Department of Medical Informatics, Erasmus University Medical Center, Rotterdam, The Netherlands; 19 Information Technology Supporting Center, Institute of Scientific and Technical Information of China, Beijing, PR China; 20 School of Economics and Management, Beijing Forestry University, Beijing, PR China; 21 Department of Computer Science and Engineering Indian institute of Technology, Patna, Bihar, India; 22 Graduate School of Information Science and Technology, Hokkaido University, Sapporo, Japan; 23 Department of Computing and Information Systems, University of Melbourne, Melbourne, Australia; 24 National ICT Australia Victoria Research Laboratory, West Melbourne, Australia; 25 Computer Science and Engineering, The Pennsylvania State University, Pennsylvania, USA; 26 Information Sciences and Technology, The Pennsylvania State University, Pennsylvania, USA; 27 Department of Health Sciences Research, Mayo College of Medicine, Rochester, USA; 28 LaSIGE, Department of Informatics, Faculty of Sciences, University of Lisbon, Lisbon, Portugal; 29 Graduate Institute of BioMedical Informatics, College of Medical Science and Technology, Taipei Medical University, Taipei, Taiwan; 30 Department of Computer Science and Information Engineering, National Central University, Taoyuan, Taiwan; 31 Department of Computer Engineering, Middle East Technical University, Ankara, Turkey; 32 Departament Ciències Mèdiques Bàsiques, Universitat de Lleida, Lleida, Spain; 33 Departament d'Informatica i Enginyeria Industrial, Univesitat de Lleida, Lleida, Spain; 34 Computer Science Department, Universidad Carlos III de Madrid, Madrid, Spain

**Keywords:** named entity recognition, BioCreative, text mining, chemical entity recognition, machine learning, chemical indexing, ChemNLP

## Abstract

The automatic extraction of chemical information from text requires the recognition of chemical entity mentions as one of its key steps. When developing supervised named entity recognition (NER) systems, the availability of a large, manually annotated text corpus is desirable. Furthermore, large corpora permit the robust evaluation and comparison of different approaches that detect chemicals in documents. We present the CHEMDNER corpus, a collection of 10,000 PubMed abstracts that contain a total of 84,355 chemical entity mentions labeled manually by expert chemistry literature curators, following annotation guidelines specifically defined for this task. The abstracts of the CHEMDNER corpus were selected to be representative for all major chemical disciplines. Each of the chemical entity mentions was manually labeled according to its structure-associated chemical entity mention (SACEM) class: abbreviation, family, formula, identifier, multiple, systematic and trivial. The difficulty and consistency of tagging chemicals in text was measured using an agreement study between annotators, obtaining a percentage agreement of 91. For a subset of the CHEMDNER corpus (the test set of 3,000 abstracts) we provide not only the Gold Standard manual annotations, but also mentions automatically detected by the 26 teams that participated in the BioCreative IV CHEMDNER chemical mention recognition task. In addition, we release the CHEMDNER silver standard corpus of automatically extracted mentions from 17,000 randomly selected PubMed abstracts. A version of the CHEMDNER corpus in the BioC format has been generated as well. We propose a standard for required minimum information about entity annotations for the construction of domain specific corpora on chemical and drug entities. The CHEMDNER corpus and annotation guidelines are available at: http://www.biocreative.org/resources/biocreative-iv/chemdner-corpus/

## Introduction

There is a pressing need to extract information of chemical compounds and drugs from the rapidly growing scientific literature [1]. Text mining and information extraction techniques are showing promising results in the biomedical domain: A range of applications have been implemented [2] to detect bio-entities [3,4] and their relations (e.g. protein-protein interactions [5], gene-disease relations [6], and protein-mutation associations [7]), or to select relevant documents for a particular topic [8]. One of the first steps required for more complex relation extraction tasks is to find mentions of the entities of interest. In the life sciences domain the entities that have attracted most attention are genes and proteins [9], while in case of more generic texts and newswire, efforts have been made to detect information units including names of persons, organizations or locations [10].

Automated techniques with the aim of detecting (tagging) mentions of named entities in text are commonly called named entity recognition (NER) systems. Although early NER taggers typically relied on hand-crafted rules, the current trend increasingly points towards the use of supervised machine learning techniques for entity recognition [10]. Such systems *learn *a statistical model to identify entity mentions by inferring which characteristics (features) distinguish them from the surrounding text. Exploited features can be the presence of certain combinations of *orthographic *features, like consecutive characters or words (n-grams), their letter case, or the presence of digits, special characters (e.g. hyphens, brackets, primes, etc.), and symbols (Greek letters, @, $, etc.). Also the ending or beginning of words (affixes) and the presence of particular terms found in a list (gazetteer) of precompiled names are often exploited by NER systems [10,11] and can help identify a word's *morphology *(inflections, gerund, pronouns, etc.). For instance, when looking at the chemical literature, it becomes clear that in case of systematic chemical names they do look quite different from common English words, mainly due to the nomenclature rules that define chemical naming standards.

Supervised methods *classify *word (token) sequences by assigning them to one of a set of predefined entity classes. For this task, they require labeled example data that commonly is split in two collections. The first collection is called the *training set*, from which the model infers its parameters. The trained model is then used to detect entity mentions in the second collection, the *test set *; This set is used to evaluate the quality of the learned model. If satisfactory, the parameterized model can then be applied to detect entities in new, unlabeled text. Therefore, labeled text is important not only to build machine learning-based entity taggers: It also can be used to evaluate the performance of any kind of NER system, regardless the underlying method used. Producing labeled data for this purpose therefore refers to the construction of properly annotated text, a so-called **corpus**. This process requires adding metadata (the annotations) to the original text according to specific *annotation guidelines*.

Over 36 corpora have been generated in the biomedical field [12] already. When the corpus contains documents with manually marked up annotations done by domain experts, they are known as *Gold Standard Corpora *(GSC). Because the manual annotation process is very laborious, lower quality corpora can be constructed by using automated techniques. A few such *Silver Standard Corpora *(SSC) have been published, too, such as the CALBC corpus [13]. Chemical (named) entities are important for chemistry, but also for other research areas such as life sciences, pharmacology, medicine, material sciences or physics. Yet, despite their wide-spread use, only few corpora with manually labeled chemical entities exist to date.

### Biology corpora with chemical entities

There are several corpora developed in the life sciences domain that include text annotations of chemical substances. A widely used and valuable resource for biomedical language processing is the *GENIA corpus *[14]. It contains a collection of PubMed abstracts annotated semantically with a variety of different entity types defined in the *GENIA Chemicals ontology*. Most of the underlying concept classes were derived from categories found in *Medical Subject Headings *(MeSH), a hierarchical terminological resource used to index PubMed abstracts [15]. The GENIA chemical concepts do correspond to a rather broad interpretation of chemicals, many of which cannot be linked to any concrete chemical entity with an associated structure. In this corpus, qualifier terms and chemical role/application terms are also annotated as chemical entities. There are no exhaustive annotation guidelines for chemical compounds underlying the GENIA corpus annotation, being essentially tailored towards biologically relevant annotations. Moreover, in GENIA, chemical entity annotations were not prepared by a chemist and chemical annotations relied mainly on human interpretation of the text and background knowledge. The *CRAFT corpus *[16] is a corpus of 97 full text biomedical articles that contains several different concept annotation types including a type consisting of chemical concepts from the ChEBI ontology [16]. This type includes chemicals, chemical groups, atoms, subatomic particles, biochemical roles and applications [17]. Annotations of the CRAFT corpus were done by biologists based on annotation guidelines that also included a set of linguistic aspects for text span markup. Chemical annotations in the CRAFT corpus were not exhaustive, being restricted mainly to the concepts covered by the ChEBI ontology. The coverage of this ontology for the chemical space published in the literature is unclear. Another hand-annotated life sciences corpus that contains chemistry-related annotations is the *PennBioIE CYP 1.0*. This corpus of 1,100 abstracts requires payment of a license fee and is focused on a rather narrow scope, the inhibition of cytochrome P450 enzymes. It includes chemicals under a semantic class called substance. This substance class is rather vaguely defined and includes proteins and other substances as well as role and functional terms. There are a few corpora that are primarily concerned with the annotation of relationships that involve chemicals, and more particularly drugs. The *EU-ADR *corpus has 300 abstracts including drug-target and drug-disease relations [18]; it was pre-annotated automatically and missed or incorrect annotations were manually corrected. With a similar scope, the *ADE corpus *contains annotations of drug-related adverse effects, covering chemicals/drugs in a therapeutic context for 3,000 abstracts. In case of the *DDI corpus*, 700 documents (both PubMed abstracts and DrugBank records [19]) were annotated for drugs and relations between them [20], while the *EDGAR corpus *(103 PubMed abstracts about cancer) also contains annotations of drugs in addition to genes and cells [21]. The *Metabolites and Enzymes corpus *[22] has annotations of metabolites, carried out on 296 abstracts on yeast metabolism. The annotation in this corpus was restricted only to those names that appeared in the context of metabolic pathways. There was also one chemistry-disease relation corpus generated from 21 US patents that contained claimed structure-activity-relationships. These patents were automatically tagged with chemistry and disease terms. The annotations process was restricted to the manual classification of the relation type existing between co-occurring terms [23].

### Chemical text corpora

As opposed to the previously introduced corpora, a number of corpora have also been described that are more focused on chemistry and chemical entities rather than on biological aspects of chemical substances. They provided important lessons for the construction of the CHEMDNER corpus. Nevertheless they also showed crucial differences in scope, used document collections, availability (both of annotation guidelines together with the resulting corpus), format and size. Early attempts to build a chemical NER systems, due to the lack of a chemical entity text corpus, explored the use of lexical resources related to chemistry derived from the UMLS Metathesaurus, which was used for training and testing various methods [24]. Wren published a machine learning method trained on the chemical ChemID database and used it to find chemical entity mentions in PubMed abstracts. Due to the lack of an evaluation text corpus he could only assess the precision on a small sample of putative chemical names extracted automatically [25]. Another publication by Zhang described the use of chemical annotations done by the indexers of the National Library of Medicine (NLM) [26] as a proxy for evaluating a chemical entity recognition system. These annotations are only done at the document level without specifying the exact entity mention offsets within the abstract. The NLM indexers annotate topic-related chemical concepts and therefore the indexing is not exhaustive. This type of annotation only reflects the understanding of the topic by the individual indexer. The document indexing was based on terms of the MeSH tree associated with chemicals (*Chemicals and Drugs *branch and supplementary concept records called *MeSH substances*). Narayanaswamy and colleagues described a small corpus of 55 abstracts selected by a keyword search (using as query *acetylates*, *acetylated *and *acetylation*) that contained also a small number of chemical names [27]. The text corpus introduced in the article describing the ChemicalTagger system consisted in 50 paragraphs from the experimental sections of full text articles selected using a keyword search related to *polymer synthesis*. It is concerned with the annotation of chemical phrases rather than on chemical entity mentions and the associated link to the annotation guidelines was not functional anymore (broken link) [28]. The ChEBI Patent Gold Standard corpus was created as a joint effort between curators of the ChEBI database and the European Patent Office [29]. It involved the annotation of chemical entities in 40 patent documents (18,061 chemical entities, 47% of them were initially linked to ChEBI records). This corpus is publicly available but more details on the annotation criteria and process were not released together with the corpus. This corpus was generated manually without using any software to create pre-annotations. An updated version of this corpus was also published to increase the initial mapping of mentions by using an updated version of the ChEBI database (53.7% of ChEBI mapped chemical entities) [30]. A recent effort carried out by both academia and commercial teams resulted in a larger corpus of 200 patents annotated with chemical information [31]. These patents were automatically pre-annotated with chemical names and human curators revised and corrected mis-identified pre-annotations and added missing chemical mentions manually. The annotation guidelines used for constructing this corpus were partially based on the annotation guidelines that we have released for the CHEMDNER corpus, as detailed later in this manuscript. A relevant contribution to the development of chemical corpora was provided by the authors of the Sciborg corpus [32,33] and the Chemistry PubMed corpus by Corbett et al. [33,34] Unfortunately neither of these two corpora are publicly available, but the underlying annotation criteria shared by both datasets had a deep impact on the annotation guidelines prepared for the CHEMDNER corpus. The Sciborg corpus consisted of 42 full text chemistry research papers annotated manually with chemical compounds while the chemistry PubMed corpus by Corbett et al. consisted in an hand-annotated corpus of 500 PubMed abstracts selected using the query '*metabolism[Mesh] AND drug AND hasabstract*'. Both corpora consisted in exhaustively annotated chemical texts done by chemists according to very detailed annotation rules (31 pages long guideline containing 93 rules, together with example cases [33]). Different annotation classes were defined to deal not only with chemical compounds but also with chemical reactions, chemical adjectives, enzymes and chemical prefixes.

A more granular annotation specifically of the chemical compound mentions was proposed for the construction of the open access Chem EVAL corpus (a.k.a. SCAI corpus), a small corpus of 100 abstracts (with 1206 chemical mentions) annotated with chemical entities [35]. Details on the actual definition and selection of chemical compound mentions were not provided together with this corpus, and the original authors stated that additional evaluation and refinement of the corpus and its guidelines is work in progress. Nevertheless this corpus proposes several types of chemical mention classes of practical relevance, which were modified and adapted for the annotation of chemical mention classes of the CHEMDNER corpus. The chemical classes proposed by them included IUPAC (systematic and semi-systematic chemical names), PART (partial IUPAC names), TRIVIAL (trivial names), ABB (abbreviations and acronyms), SUM (sum formula, atoms, molecules, SMILES and InChI) and FAMILY (chemical family names). The distinction between TRIVIAL and IUPAC was an arbitrary decision according to the name length: names with one word were considered as TRIVIAL, while multi-word systematic and semi-systematic names were labeled as IUPAC.

### Chemical names and challenges for NER

To be able to implement and compare the performance of chemical NER systems the availability of large enough manually tagged text corpora is a key requisite. It is thus not surprising that a comparative evaluation effort for this topic had not been carried out prior to the release of the CHEMDNER corpus. The intrinsic difficulty in defining annotation guidelines of what actually constitutes a chemical compound that can be linked to structural information was the main difficulty in constructing the CHEMDNER corpus. Although the International Union of Pure and Applied Chemistry (IUPAC) has defined a set of rules for the chemical nomenclature, those naming standards are not sufficiently followed in practice when examining the scientific literature [36]. Chemistry is a research discipline with a considerable degree of specialization that can explain the encountered variability of language use between its sub-disciplines. Moreover chemical entities are also studied in publications from other disciplines such as medicine, biology and pharmacology. Thus a virtually arbitrary number of language expressions may be found in the literature to refer to chemical compounds. This variability can be explained by the use of aliases, e.g. different synonyms used for the same entity. For instance the antidiabetic and anti-inflammatory drug 'troglitazone' also has the brand name 'Rezulin', while its systematic (IUPAC) name would be '(*RS*)−5−(4−[(6 −*hydroxy*−2,5,7,8−*tetramethylchroman*−2−*yl*)*methoxy*]*benzyl*)*thiazolidine*−2,4−*dione*'. Variability can also be simply due to alternative typographical expressions referring to the same chemical. The problem of variability has a negative impact on i) the resulting recall of NER systems (fraction of the total entities mentioned in text that are recognized by a system) and ii) the feasibility to map all the various alternative compound mentions to its corresponding unique canonical chemical structure.

Ambiguity, the fact that a given word can correspond to a chemical entity or to some other concept depending on the context of the mention, also poses difficulties for labeling text with chemical entities. A source of ambiguity for chemical entities is the heavy use of acronyms, abbreviations, short chemical formula and certain trivial names used in the literature. Additionally, a few common English words such as gold, lead and iron are also a source of ambiguity for NER systems. The following list summarizes some of the challenges related to chemical entity mention annotation and automatic recognition.

• Difficulties in defining what a chemical entity is.

• The official IUPAC nomenclature guidelines are only partially followed in practice in the literature.

• Chemical compounds/drugs often have many synonyms or aliases (e.g. systematic names, trivial names and abbreviations referring to the same entity).

• Existence of hybrid chemical mentions (e.g. mentions that are partially systematic and trivial).

• Chemical compounds are ambiguous with respect to other entities or terms (in particular abbreviations and short formula).

• Existence of naming variation: typographical variants (alternating uses of hyphens, brackets, spacing, etc.) and alternative word order.

• New chemical compound are discovered and described in papers every day (novel chemical names).

• Definition of both chemical entity mention boundaries and word tokenization is complicated.

For the successful detection of chemical entity mentions, tools need to be able to cope as much as possible with these difficulties.

### BioCreative task on chemical entity recognition

Chemical entities of practical importance are those that can be ultimately linked to chemical structure information, rather than general vague chemical concepts. Being able to associate a given chemical compound name to a chemical structure was the central annotation criteria followed for the construction of the CHEMDNER corpus. The details on the construction of the CHEMDNER corpus will be provided in the following sections. To demonstrate its utility, the CHEMDNER corpus was used as the dataset to train and evaluate chemical NER systems that participated in a task posed at the fourth BioCreative community challenge [11]. The BioCreative challenges are an ongoing effort to promote the evaluation and development of text mining and natural language processing software for the life sciences community [37]. Carrying out this task within the organization of BioCreative was especially useful due to the previous experiences of this community with related bio-medical NER tasks (the Gene Mention recognition tasks of BioCreative I and II [38,39], as well as the Gene Normalization tasks [40]).

## Methods

The construction of the CHEMDNER corpus started with the definition of the overall *annotation goal *together with an exhaustive revision of previous work done on annotation of chemical entities as well as named entities in the biomedical and other domains. The aim while defining the chemical entities annotated for the CHEMDNER corpus was to capture only those types of mentions that are practically relevant. The common characteristic among all the chemical mention types used for the CHEMDNER corpus was that they could be associated to chemical structure information with at least a certain degree of reliability. We consider this aspect of crucial practical relevance. The annotation carried out for the CHEMDNER corpus was only exhaustive for this particular type of chemical mention, which we named *Structure Associated Chemical Entity Mentions *(*SACEMs*). For example 'nitric oxide', 'resveratrol' or 'malondialdehyde' would constitute example cases of SACEMs, while general chemical concepts like 'inactivator' or 'pigment', biological roles like 'hormone', 'antibiotic' or 'metabolite' and reactivity roles like 'nucleophile' or 'chelator' do not qualify as SACEMs. This implies that other types of mentions of chemicals and substances were not annotated. In order to construct the CHEMDNER corpus we examined several critical aspects that we thought influence the corpus quality.

• Corpus selection and sampling.

• Annotation guidelines and their corpus-driven refinements.

• Entity annotation granularity.

• Human annotator expertise and training.

• Annotation tools and interface.

• Annotation consistency and definition of upper and lower performance boundaries to be expected by automated systems.

• Corpus format and availability.

**Figure 1:**
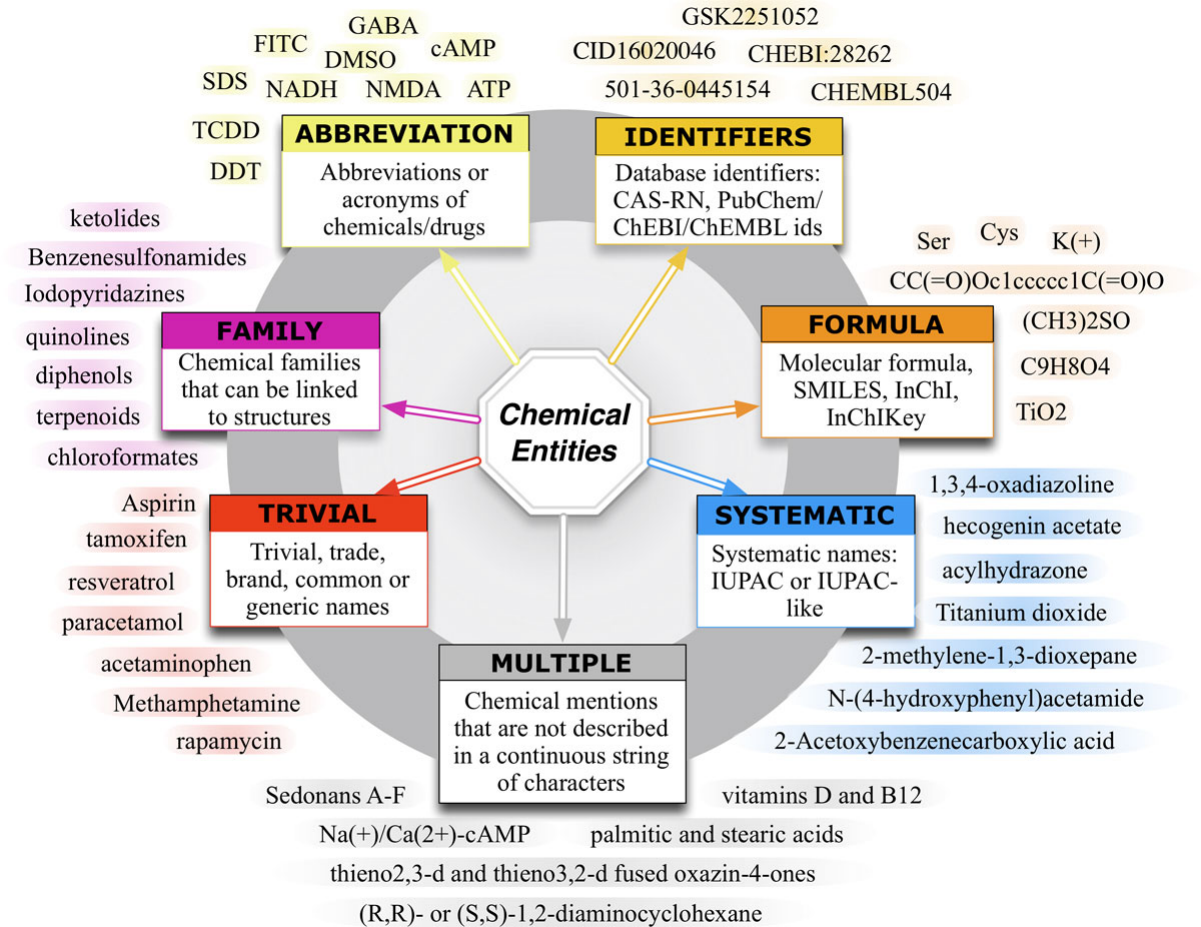
**CHEMDNER chemical entity mention classification chart and examples**.

From an initial examination of SACEM mentions it was clear that chemicals in text appeared in various forms. We therefore proposed a more granular annotation schema that covered the most important types of chemical mentions that can be found in the literature. We introduced seven classes of SACEMs, inspired by previously introduced chemical mention types [35]. Figure 1 provides an overview of the chemical mention classes together with a short description and example cases. When defining these classes, the following issues were contemplated: *semantically relevant aspects *of chemicals, the usefulness of the class information for subsequent NER detection methods (*detection strategies*) and their implication in chemical structure normalization of the mentions (*normalization strategies*). Depending on the chemical mention class, different strategies for linking mentions to chemical structures have to be used (e.g. dictionary-based strategy for trivial names or name to structure conversion software for systematic names). In the CHEMDNER corpus, the following *CEM classes *were introduced: SYSTEMATIC, IDENTIFIERS, FORMULA, TRIVIAL, ABBREVIATION, FAMILY and MULTIPLE.

### Document selection and sampling

An often-underestimated aspect when constructing text corpora is the initial selection of the documents that should be annotated. Using a keyword based article selection has the risk of generating a rather narrow or biased dataset, especially when the aim is named entity recognition. In order to make sure that the NER tools developed on the CHEMDNER corpus will generalize well on any chemistry-related document we used a careful selection strategy. The used CHEMDNER document set had to be representative and balanced in order to reflect the kind of documents that might mention the entity of interest. In case of chemical entities it is essential to cover articles that show sufficient diversity of the kind of mentions expected to emerge across various chemical disciplines. The articles should have enough cases of systematic names, common or generic names of compounds and drugs, trade names, identifiers, acronyms, reference numbers of compounds and even formulas. In case of the CHEMDNER corpus the document selection criteria took into account primarily the scientific discipline of the journals and publication dates. The following steps were used to select abstracts for the CHEMDNER corpus.

**Step 1: **Selection based on subject categories from the ISI Web of Knowledge relevant to various chemistry-related disciplines: BIOCHEMISTRY & MOLECULAR BIOLOGY; APPLIED CHEMISTRY; MEDICINAL CHEMISTRY; MULTIDISCIPLINARY CHEMISTRY; ORGANIC CHEMISTRY; PHYSICAL CHEMISTRY; ENDOCRINOLOGY & METABOLISM; CHEMICAL ENGINEERING; POLYMER SCIENCE; PHARMACOLOGY & PHARMACY and TOXICOLOGY.

**Step 2: **Selection of the top 100 journals for each category based on the journal impact factor.

**Step 3: **Selection of journals that had at least 100 articles.

**Step 4: **Selection of articles that were published in 2013 in English, with abstracts and links to full text articles in the PubMed database.

**Step 5: **Selection of articles that belonged to the various subject categories.

**Step 6: **Randomization of the abstracts and selection of 10,000 records

**Step 7: **Splitting into three datasets: 3500 (training set), 3500 (development set) and 3000 (test set) abstracts.

The CHEMDNER corpus therefore contains representative articles for a range of chemistry-related fields. It is sufficiently large to cover the most relevant mention types and naming variability that are encountered in the scientific literature, allowing both to generate a predictive model and train an NER recognizer on a subset of abstracts as well as evaluate the performance on a distinct test collection. We selected recent publications to make sure that the corpus would be useful for the detection of chemical entities in new abstracts as soon as they get published. It also covers journals with an high impact in the field based on its impact factor and the number of published articles by that journal.

### Annotation guidelines

Surprisingly there are many manually annotated text corpora that are not distributed together with detailed guidelines describing how the annotations were generated. Such *black box corpora *have the disadvantage that they *cannot be extended*, it is *impossible to compare them *in a meaningful way to other corpora and it is *unclear how to deal with potential causes of inconsistencies and annotation errors*. Annotation guidelines should specify the necessary instructions to identify the text elements that should be tagged (and those that shouldn't be tagged) and how to assign them to its corresponding entity class. At a general level they do represent the instructions on how the annotation schema should be applied to the actual text data that will be labeled.

Three important things had to be addressed in the annotation guidelines: (a) *what to label*, (b) the *mention boundaries *of those labels, and (c) how to *classify those mentions *into chemical mention categories.

To create high quality guidelines that fit the annotation task required a *multi-step iterative process*: starting from an initial guideline draft until clear and refined guidelines were obtained. In case of the CHEMDNER corpus, to define the *text-bound annotations *of chemical mentions was not trivial. It required a deep knowledge of chemistry, supported with consultation of external knowledge sources in case of doubt. The guidelines were prepared by chemists with feedback of trained literature curators also with a Ph.D. in chemistry. In order to label SACEMs mentions, a set of annotation rules were defined. These rules were initially adapted by reviewing the annotation guidelines for chemicals from the manual prepared by Corbett et al. [33] (version 6.0, 2007). The CHEMDNER annotation rules had several important modifications: (1) only chemical nouns (and specific adjectives, treated as nouns) were considered (not reactions, prefixes or enzymes); (2) the number of original rules was reduced; (3) rules were grouped as positive, negative, orthography and multi-word rules. In case of the multi-word rules some simplifications were done, making less error-prone to human interpretation.

Very general chemical concepts (non-structural or non-specific chemical nouns), adjectives, verbs and other terms (reactions, enzymes) that cannot be associated directly to a chemical structure were excluded from the annotation process. SACEMs for this task had to refer to names of specific chemicals, specific classes of chemicals or fragments of specific chemicals. General chemical concepts, proteins, lipids and macromolecular biochemicals were excluded from the annotation. Therefore genes, proteins and protein-like molecules (above 15 amino acids in length) were not annotated. Chemical concepts were labeled solely if they provided concrete structural information. Relevant and intuitive examples cases (*rule instantiation examples*) were provided in the guidelines when necessary to represent a specific annotation rule, to make it easier to understand and apply them. Although chemical intuition of the annotators was important for defining the annotation guidelines we did not require any specific linguistic background knowledge.

#### Stage 1 -- Pre-annotation guideline discussion round

At the very beginning, before a sample set was annotated, the annotators revised the guidelines and posed questions to improve the guidelines in a first refinement round. At this stage, the annotation specifications were reformulated if ambiguities or inconsistencies were detected.

#### Stage 2 -- pilot annotation guideline testing and refinement

Then, the initial set of rules was then tested in practice by using them to annotate a small sample of abstracts (the *seed corpus*). The seed corpus was annotated by curators to examine the suitability of the stage 1 guidelines. During this pilot annotation experiment: we estimated the required annotation time effort; refined iteratively the guidelines (to make them more precise and easier to follow, resolving cases of under-specification); learned how to use the annotation interface and how it fitted the needs required for annotating the mentions according to the guidelines.

#### Stage 3 -- corpus annotation

The last step consisted in the annotation of the training, development and test set. During the corpus annotation stage, the guidelines were refined when novel, previously unspecified ambiguities were encountered. These ambiguities were resolved through direct feedback with the experts that constructed the guidelines. Moreover new example cases were added to the guidelines.

The CHEMDNER annotation guidelines are publicly available together with the corpus at [41]. In an attempt to facilitate its reading, the guidelines are structured according to six different types of rules, while trying to keep them as comprehensive as possible:

*General rules*: rules that clarify the use of external knowledge sources and how to deal with unclear mentions.

*Positive rules*: rules that specify which chemical entity mentions should be labeled.

*Negative rules*: rules that specify which kind of mentions should not be tagged.

*Class rules*: specifications for the manual assignment to the corresponding CEM classes, including hybrid names.

*Orthography and grammar rules*: rules for defining consistently the entity mention boundaries, dealing for instance with whitespaces, mis-spellings, flanking characters, commas, brackets, etc.

*Multi-word entity rules*: rules defining labeling criteria for multi-word chemical entities.

The CHEMDNER annotation guidelines, including the example cases are 21 pages in total. One of the most important and difficult issues when defining the guidelines was to establish what constitutes a chemical mention and what does not. A single, particular chemical compound assignable to a chemical structure can be easily recognized by a chemist. The problem arises for general terms comprising several structurally diverse chemical compounds and for which the mention intrinsically provides a general notion of structural class. For example, the term 'Alkaloid' refers to a group of naturally occurring chemical compounds that mostly contain basic nitrogen atoms. From a practical viewpoint, it would be worthy to tag this SACEM as a FAMILY because an end-user could be interested in recognizing this family of compounds in a given biomedical context. However, strictly talking, a single simple Markush formula can not be assigned to this class. In an attempt to homogenize the criteria, an exemplary list (probably expandable in future releases) was provided in the guidelines. As the number of potential mentions of this kind is not really high compared to the rest of mentions, this should not strongly affect the final conclusions of the task. An additional problem with these mentions is that most of them are natural products commonly found in living organisms, so the frontier between chemistry and biology is not easily traceable. As mentioned, a limit on the size of the peptides, sacharids, nucleotides and lipids was imposed as a solution for these small biochemicals. A second problematic issue was establishing how to deal with the adjectives. Adjectives preceding valid SACEMs that add more precise information on the chemical entity were annotated. Finally, the exact assignation of the mentions to the FAMILY class was controversial in some cases and exemplary cases were added during the iterative refinement. For example, synthetic polymers consisting of an undefined number of monomers were detected during the annotation and incorporated to this class.

### Annotation process and interface

**Figure 2:**
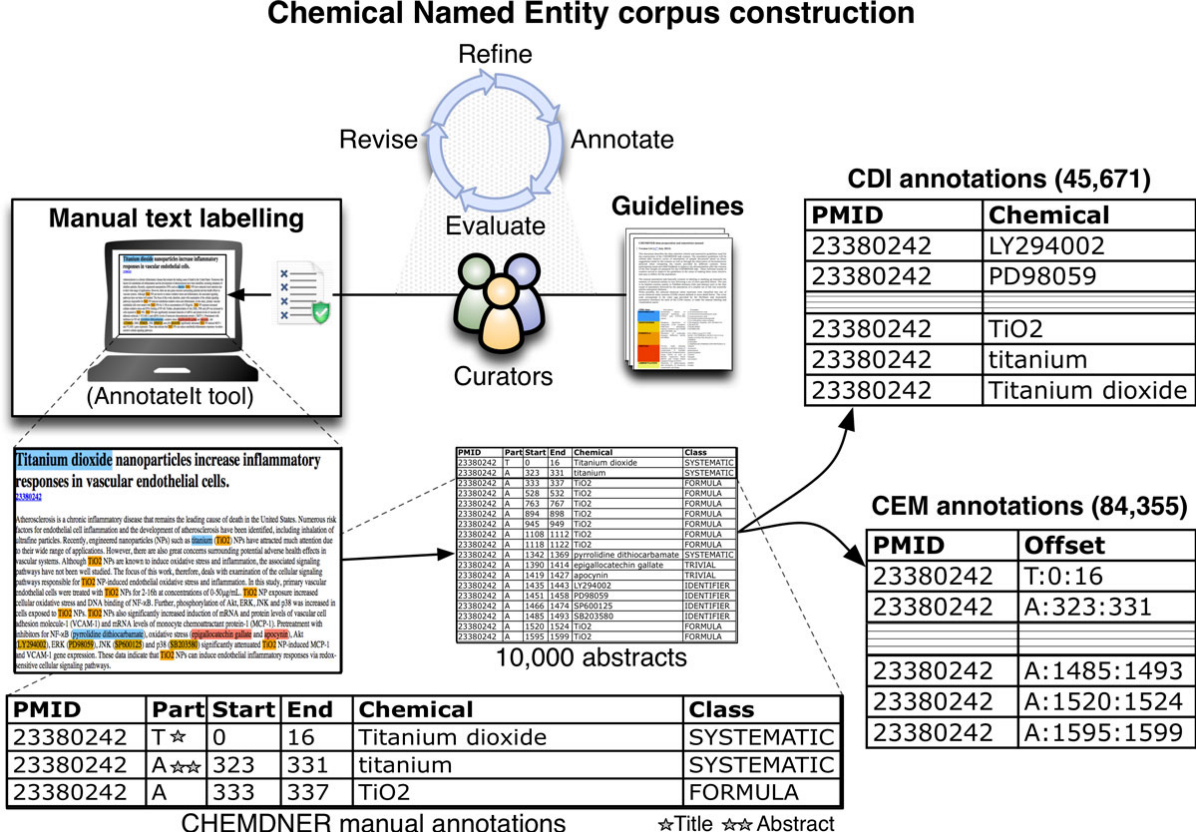
**Left side: Overview of the manual CHEMDNER corpus annotation process**. Right side and bottom: Annotation examples for the Chemical Document Indexing (CDI) and Chemical Entity Mention (CEM) task.

It is important to define the minimal *curator selection criteria*, i.e. the skills that are required to carry out the annotation task and to make sure that the curators have a suitable background and are familiar with the annotation of literature data. A prerequisite for the manual annotation for the CHEMDNER corpus was that annotators had to have a background in chemistry to guarantee that the annotations are correct. The group of curators used for preparing the annotations was composed mainly of organic chemistry postgraduates with an average experience of 3-4 years in annotation of chemical names and chemical structures. The curators were trained to mark up the text according to the labels specified in the guidelines. The raw text was not tokenized prior to the annotation and only the title was distinguished from the PubMed abstract. The selection of text spans was done at the character level, we did not allow nested annotations and distinct entity mentions should not overlap. Each text span was selected according to the annotation guidelines and classified manually into one of the CEM classes. Figure 2 provides a very general flowchart of the CHEMDNER annotation process resulting in the annotations used for the BioCreative CHEMDNER task. The annotation modus operandi itself relied on the chemical background knowledge of the curators (and comprehension of the guidelines) during the labeling of the chemical entity mentions. We recommended the curators to consult existing chemical knowledgebases in case of doubts. They could crosscheck information from reference sources such as Wikipedia, and chemical databases (ChEBI, DrugBank, etc.) or even carry out online searches to make sure that the annotations were compliant with the guidelines. Annotators knew that the text collection corresponded to PubMed abstracts and they were provided with pointers to the original PubMed records. No additional meta-data or automatically pre-tagged text was provided. We initially experimented with a *pre-tagging strategy *using a specially adapted version of the MyMiner system [42] that included the option of pre-tagging the text with Oscar4 and then manually correcting the labels. The automatic pre-tagging strategy had limitations in terms of performance and had the potential of biasing the curation results. Moreover, as we also requested the classification of mentions into one of the seven chemical mention classes, we finally chose to use an exclusively manual annotation approach. For manually tagging a large collection of abstracts distributed across multiple curators it is crucial to test suitable annotation infrastructures that are scalable and that can efficiently manage and visualize the generated annotations. Thus, together with distributing the guidelines we made sure to provide efficient access to a suitable text curation tool. Therefore we required that the graphical user interface allows labeling of text efficiently and consistently. We explored alternative ways on how to present the documents to the annotators in a way that is supported by existing annotation tools. In addition to the MyMiner tool, the systems Brat and Knowtator were examined [12]. Finally we decided to adapt the AnnotateIt tool [43] as the curation application for the construction of the CHEMDNER corpus. It doesn't require local installation on the curators side. It can be used through a web-browser and it makes the annotation process as easy and fast as possible. The *annotation tool selection criteria *that we examined for choosing this system are as follows: (1) It should be fast in loading previous annotations and adding new labels, (2) it should be scalable for the annotation of data large collections (10,000 abstracts), (3) it should make sure that the annotations were not lost due to time-outs etc., (4) it should allow that the annotations could be created using an intuitive web-browser interface and (5) it should accurately capture the entities' Unicode character offsets. Figure 2 contains an example screenshot of the interface used to generate the manual annotations for the CHEMDNER corpus. The curators were provided with a short demo video illustrating how the interface worked. A color code schema was defined for tagging and visualizing the different SACEM classes. We provided recommendations specifying browser settings that should be used during the annotation process. The input abstracts were previously randomized to avoid that the ordering of abstracts could have an effect on the curation process. Annotation was carried out in annotation batches of 100 abstracts each.

### Annotation format

In principle, the information represented in annotated textual data can be represented in various alternative formats reflecting how the annotations look like. For choosing the annotation format of the CHEMDNER corpus, several criteria were important. First of all, the format should be easy to use for building NER systems, thus it should be simple and easy to modify. There was a clear separation of the *entity annotation format *and the *exchange (dump) format *of the released CHEMDNER corpus. This means that we kept the annotations separate from the actual text (the information on the location of the entity mentions is stored in a different file from the actual raw text). We used a *standoff annotation *format by specifying in a separate file the character location. Using *character offsets *instead of token location was particularly important for the CHEMDNER corpus because it makes it easier for the corpus consumers to use their own text tokenization strategy. We avoided using a complicated XML schema for the initial baseline release. We examined some basic recommendations provided by the Linguistic Annotation Framework (LAF) for data distribution [44]. All records used for the CHEMDNER corpus were distributed as plain text, UTF8-encoded PubMed abstracts in a tab-separated format with the following three columns: article identifier (PMID, PubMed identifier), title of the article, and abstract of the article. The baseline entity annotation file had a tab-separated format with columns corresponding to the article identifier, the part of the document processed (T: title, A: abstract), the start and end characters offsets of the chemical, the text string of the chemical entity mention and the corresponding chemical entity mention class. Example cases of the entity annotation file can be seen on Figure 2. The *task annotation files *were derived from the entity annotation file, one for the CEM task and one for the CDI task. In addition to this simple annotation format we have recently generated a version of the CHEMDNER corpus using an alternative format, the widely used *BioC *format [45]. The BioCXML version of the CHEMDNER corpus [41] was checked to make sure that the used XML was valid, both with respect to XML itself and the BioC DTD. The Python script to convert the flat-files of the CHEMDNER tab-separated format into the BioC format was released together with the corpus.

## Results

### CHEMDNER corpus overview

The CHEMDNER corpus is currently the largest chemical entity corpus annotated with a high degree of granularity for PubMed abstracts. A detailed summary of the total number of generated annotations of the entire CHEMDNER corpus as well as divisions according to each of the three corpus subsets (training, development and test set) can be seen in table 1. The CHEMDNER corpus contains a total of *84,355 *manual chemical mention annotations; corresponding to *19,805 *unique chemical name strings extracted from 10,000 exhaustively examined abstracts. Although the majority of the abstracts did contain at least a single chemical mention (a total of 8,301 abstracts), this table also shows that a fraction of the abstracts did not have any chemical mention at all. This smaller subset can be used as a true negative dataset of abstracts that do not mention SACEMs. Until now, such a true negative dataset for chemical entity mentions was missing. This table also shows that the annotation density across the various datasets is coherent and this in turn reflects that the CHEMDNER corpus is balanced and that overall the three subsets have a comparable (relative) number of chemical mentions. The used abstracts were derived from a total of 203 different journals from heterogeneous chemically related topics (see the subsection on Document selection and sampling). When examining the annotations according to the chemical mention classes, as shown in the lower part in table 1 the quantitative importance of two chemical mention classes becomes obvious, namely of the mention classes TRIVIAL (30.36%) and SYSTEMATIC (22.69%). These two classes make up more than half of all the annotations. It seems that the overall frequency of ABBREVIATION (15.55%), FORMULA (14.26%) and FAMILY (14.15%) is similar. Mentions of chemical identifiers (2.16%) and of the type MULTIPLE (0.70%) are quite infrequent. One common baseline strategy for entity recognition consists in tagging those entities in the test set that were previously contained in the list of chemicals of the training collection. Such an analysis also helps to illustrate the diversity and representativeness of the used data collections and examines basic aspects of the corpus characteristics. The *vocabulary transfer *is the proportion of entities (without repetition) that appear both in the training/development set as well as in the test corpus. This value is often taken as the *lower boundary of the recall *that can be expected from NER systems. In case of the CHEMDNER dataset, the vocabulary transfer was of 36.34% when uniting both the training and development set names before comparing them to the test set entity list. It was 27.77% when using only the names from the training set, and 27.70% when using only those from the development set.

**Table 1:** CHEMDNER corpus overview.

	Training set	Development set	Test set	Entire corpus
Abstracts	3,500	3,500	3,000	10,000
Nr. characters	4,883,753	4,864,558	4,199,068	13,947,379
Nr. tokens	770,855	766,331	662,571	2,199,757
Abstracts with SACEM	2,916	2,907	2,478	8,301
Nr. mentions	29,478	29,526	25,351	84,355
Nr. chemicals	8,520	8,677	7,563	19,805
Nr. journals	193	188	188	203

TRIVIAL	8,832	8,970	7,808	25,610
SYSTEMATIC	6,656	6,816	5,666	19,138
ABBREVIATION	4,538	4,521	4059	13,118
FORMULA	4,448	4,137	3,443	12,028
FAMILY	4,090	4,223	3,622	11,935
IDENTIFIER	672	639	513	1,824
MULTIPLE	202	188	199	589
NO CLASS	40	32	41	113

This table provides an overview of the CHEMDNER corpus in terms of the number of manually revised abstracts (Abstracts) with their total sizes as number of *characters *and *tokens*, the number of abstracts containing at least one chemical entity mention (Abstracts with CEM), the number of annotated *mentions *of chemical entities, the number of unique *chemicals *annotated (the non-redundant list of mentions) and the number of corresponding *journals *for the annotated abstracts. The number of mentions for each CHEMDNER entity class (see Figure 1) is provided for each set and the entire corpus in the lower half of the table.

**Figure 3:**
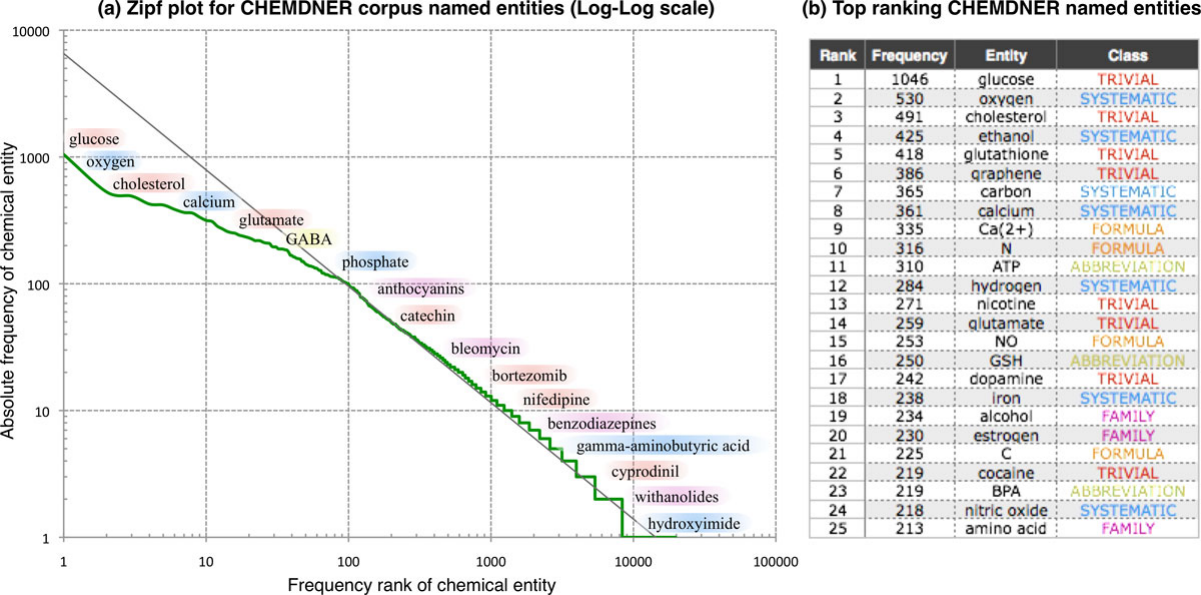
**Chemical entity frequency**. (A) Zipf plot of all chemical entities in the CHEMDNER corpus. (b) Most frequent chemical mentions of the CHEMDNER corpus. Note: The annotation guidelines specified a small stop list of chemicals that were not annotated.

In order to get a general idea on what the CHEMDNER corpus contains we carried out a simple *statistical corpus analytics *to summarize the corpus content. Figure 3 shows the statistical profile of the chemical entities contained in the CHEMDNER corpus by examining the distribution of the chemical mentions. It illustrates the CHEMDNER *corpus rank/frequency profile*, reflecting the relation between chemical entity mention frequency and the corresponding entity rank when ordering chemicals according to the resulting absolute frequency. The entity frequencies were calculated by counting the number of times a chemical entity string is found in the corpus. This plot is coherent with statistical corpus characteristics observed for token frequencies of other corpora, showing the typical behavior that corpora have commonly an uneven distribution of word types. We examined what chemical entities are most frequently used in the corpus. Part (b) of this figure provides example cases of the top frequent chemical entity names annotated in the CHEMDNER corpus. The vast majority of chemicals in the corpus had a very low frequency, and only few entities (e.g. glucose or oxygen) did have a high number of mentions. Over 72% of the chemical entities were mentioned only one or two times in the corpus. One particularity of chemical compound mentions, which differentiates it from almost any other entity type is length. Chemical compound names, especially in case of systematic names, can be particularly long. The longest chemical mention of the CHEMDNER corpus was a 349 characters long systematic name. The mean chemical mention length was 10.01 characters (median 8). There were considerable differences in length (and also character composition) between the various chemical mention classes. Mentions of type MULTIPLE were very long (mean: 27.85, median: 24 characters) because they basically corresponded to mentions of several compounds. Also systematic chemical mentions were rather long (mean: 15.48, median: 11). The other classes did all have shorter mentions: FAMILY (mean: 13.19, median: 10) TRIVIAL (mean: 10.06, median: 10), IDENTIFIER (mean: 7.25, median: 7) FORMULA (mean: 4.33, median: 3) and ABBREVIATION (mean: 3.90, median:3). Note that in case of the abbreviations, only cases of at least 3 characters were annotated according to the annotation rules.

### Corpus inter-annotator agreement and harmonization

The comparison of independent manual labels constructed for the same documents by different individuals can provide important insights on the quality of the corpus and guidelines, it is an essential element of the construction of Gold Standard corpora. It helps to assess how well the annotation task was defined; it shows how curators compare to each other and determines if the interpretation of the instructions were followed consistently. This means that the *inter-annotator agreement *(*IAA*) score allows assessing how accurate the annotations can be done by several annotators and scoring the task reproducibility. Future extensions of a corpus using the same guidelines should result in comparable inter-annotator agreement results. If the score is high, the task is well defined and the annotations are consistent. The simplest IAA score is the percentage agreement between experts. The IAA analysis of the CHEMDNER corpus was conducted using a random sample of 100 abstracts chosen from the entire dataset, asking the curators to annotate the data set independently. The result of the IAA study constitutes a sort of upper boundary for the expected automated prediction performance. An inter-annotator agreement of 91% was obtained when exact matching of the chemical mentions was used without considering the label of the SACEM classes. When the SACEM class annotation of the mentions was also considered, the IAA was of 85.26%. Manual inspection of the conflicting annotations showed that the main source of discrepancies were missed annotations by either one or the other annotator and not true annotation errors or differences in the mention boundary definition. This is in line with previously published studies, describing as one common source of disagreement between manual entity annotations that some mentions were missed by the curators while scanning over the document [33].

To make sure that during the annotation process the amount of missed chemical mentions was marginal, in addition to the main annotation team that prepared the CHEMDNER corpus, a second group of additional curators annotated the test set abstracts. These abstracts were used to score the automated mention predictions during the CHEMDNER task, and it was therefore particularly important that these annotations were complete and correct. We collected all the conflicting annotations between the two curator teams, consisting in those mentions that were only annotated by a single team. To harmonize those conflicting annotations, they were presented to the main curation group for a second round of manual revision. The entire abstract of those conflicting cases was revised to resolve the annotation discrepancies within their context. The curators provided written decisions of inclusion, exclusion or changes related to the conflicting chemical mentions together with comments explaining their decision for more complicated cases. The annotation guideline developers inspected the list of entity revisions for final approval. Written discussions were done on unclear cases that required further refinements (or additional example cases) to be included in the annotation guidelines. We relied primarily on the annotations of the main annotator team because these curators had a higher degree of experience in this task and they did provide active feedback for the refinement of the annotation guidelines. The results of the *corpus harmonization *process was that 1,185 annotations were added to the original 24, 671 test set annotations (4.08%) while 505 (2.05%) where removed, obtaining the final harmonized test set of 25,351 annotations. We performed a mention class label revision (SACEM *class label harmonization*) on the entire CHEMDNER corpus. For potentially inconsistent cases where a given chemical name was annotated in some cases as one SACEM class and in other cases as another SACEM class, the chemical entities and their SACEM class labels were manually inspected and corrected. Finally, an automatic revision of annotations was done to cross check the mention boundaries, trimming whitespace characters, and ensuring their technical coherence with the annotation rules.

A common mismatch between annotators was related to issues on how to deal with non-essential parts of the chemical name, especially concerning general modifiers (e.g. 'substituted') inside the chemical name. These modifiers should be retained whereas in some wrong cases (e.g. 'Fluorophenyl substituted 3,3'-diindolylmethane') the mention was incorrectly splitted. Closely related to this, many mismatches between annotators were detected due to a heavy trend to over split the chemical mentions into different SACEMs, especially in the case of FORMULA and MULTIPLE classes. The main variability between annotators in the SACEM class assignment was found for hybrid mentions comprising a combination of different sub-parts of the mention (typically systematic nomenclature, formula and abbreviations). A hierarchical assignation scheme was defined in the guidelines, so that the curator should label the mention according to the ranking provided for the SACEM: SYSTEMATIC has preference over the rest of SACEMs, FORMULA over TRIVIAL and so on. Some examples for the different combinations were initially provided in the guidelines and a few more were incorporated during the iterative guidelines refinement process. We think that the hierarchical SACEM class assignment guidelines require further improvements. Dealing with the FAMILY class could also be improved. For example, general FORMULA involving more than a single compound were wrongly assigned to the FORMULA class instead of the FAMILY class.

### Chemical disciplines CHEMDNER subsets

The CHEMDNER corpus contains articles from various chemistry-related disciplines. Some journals used during the selection process did correspond to multiple ISI Web of Knowledge subjects. This means that the systems trained and evaluated using the CHEMDNER corpus should in principle generalize well across the main chemistry disciplines. Nevertheless, there are scenarios were it is useful to have a system tailored specifically for a narrower *chemical application area *or discipline in addition to a *general chemical tagger*. Each chemical discipline is characterized by certain particularities in terms of sub-language and differences in chemical entity mentions and mention classes. We have provided the classification of each article into various chemical disciplines, enabling the possiblity to create the following CHEMDNER *domain-specific subsets*: BIOCHEMISTRY, APPLIED CHEMISTRY, MEDICINAL CHEMISTRY, MULTIDISCIPLINARY CHEMISTRY, ORGANIC CHEMISTRY, PHYSICAL CHEMISTRY, ENDOCRINOLOGY, CHEMICAL ENGINEERING, PHARMACOLOGY, POLYMER SCIENCE and TOXICOLOGY. These subsets were based on the ISI Web of Knowledge subjects. Although a manual revision of the CHEMDNER journals could allow a more accurate journal categorization, the subject categories used here are still useful to enable the examination of the performance of various taggers specifically for particular chemical disciplines. Some of these subsets are large enough to serve as training and test set to generate *sub-domain specific chemical entity taggers*. Table 2 provides an overview of the number of articles and annotations of each subset. It also highlights general differences between the kinds of chemical mentions used by researches from the various chemical fields. For instance, in polymer science and toxicology the use of abbreviations is very frequent while the use of chemical formula is common in the physical chemistry literature. In organic chemistry and pharmacology the use of chemical identifiers and trivial names seems to be more extended than in other domains.

**Table 2:** CHEMDNER abstracts, split into chemical disciplines (subject categories, first column; MULTIDISCIPL. CHEM.: Multidisciplinary Chemistry).

Chem. subject categories	Abstracts	Mentions	AB	FA	FO	ID	MU	NO	SY	TR
PHARMACOLOGY	1,983	23,368	18.81	10.54	6.42	4.93	0.64	0.29	17.28	41.09
MEDICINAL CHEMISTRY	1,957	17,543	10.00	21.11	8.00	2.10	1.56	0.12	25.88	31.23
ORGANIC CHEMISTRY	1,893	22,622	18.77	10.56	6.56	5.00	0.63	0.30	17.43	40.74
TOXICOLOGY	1,664	21,608	20.82	10.59	14.16	1.35	0.46	0.13	22.68	29.81
MULTIDISCIPL. CHEM.	1,217	11,892	14.38	12.15	27.97	0.52	0.55	0.13	25.62	18.67
PHYSICAL CHEMISTRY	997	9,682	12.14	9.81	36.39	0.27	0.43	0.15	27.57	13.24
BIOCHEMISTRY	879	6,503	18.75	16.55	14.24	1.12	0.34	0.11	23.17	25.73
APPLIED CHEMISTRY	843	7,759	8.48	24.45	7.71	0.17	1.37	0.10	24.99	32.74
ENDOCRINOLOGY	652	5,484	14.66	16.01	9.87	1.33	0.15	0.15	20.13	37.71
POLYMER SCIENCE	232	1,999	33.82	17.26	6.50	0.05	0.10	0.00	25.86	16.41
CHEMICAL ENGINEERING	3	42	0.00	0.00	38.10	0.00	0.00	0.00	61.90	0.00

**Abstracts**: The number of abstracts associated with that category in the CHEMDNER corpus. **Mentions**: The total number of chemical entity mentions in the abstracts of that category. Remaining columns: The values provided for the different SACEM classes correspond to the percentage of mentions in that category; AB: ABBREVIATION, FA: FAMILY, FO: FORMULA, ID: IDENTIFIER, MU: MULTIPLE, NO: NO CLASS, SY: SYSTEMATIC, TR: TRIVIAL.

### CHEMDNER corpus test set predictions

Most of the existing biomedical corpora are not distributed together with the results of automated systems predictions trained or tested using these datasets. This makes it impossible to do a more *exhaustive and detailed analysis of the differences between various methods at the level of concrete annotations*. When a corpus was used to generate multiple predictions, for instance by different teams of a community challenge, it is interesting to check various run combinations or construct an ensemble systems with improved performance over the best single run. The competitive performance of ensemble systems has been demonstrated for instance for the recognition of gene mentions [46] or the detection of protein interactions [47], showing in some cases that even low scoring runs can positively contribute to the ensemble system performance. Moreover, we think that the release of corpus predictions is useful to examine more difficult or easier cases and to detect potential annotation errors when examining consensus predictions generated by multiple systems. We have included with the CHEMDNER release the predictions generated by participating systems for the BioCreative CHEMDNER task [11] with the aim of keeping the research on this topic alive and facilitate the improvement of chemical taggers and the corpus annotations. A general characterization of methods, resources, features and performance of the various systems can be found in the CHEMDNER overview paper published in this same special issue [11]. Extra details on each of the methods can be found for a subset of competitive approaches in the systems description papers of this special issue, the CHEMDNER evaluation workshop proceedings [48] and in Additional file 1. The best F-score obtained for the chemical mention recognition by a single run was 87.39%. For the 3,000 test set abstracts, 26 teams returned 105 different runs, containing a total of 2,565,430 chemical mention predictions. Additional file 2 shows the clustering of all runs in terms of how similar the predictions between the runs are. The mean number of predictions for the test set was 24,432.67 (standard deviation of 12,429.69), corresponding to an average of 8.14 predicted mentions per abstract. When looking at fraction of abstracts that had manually annotated mentions (82.6%) and the average number of abstracts predicted to have at least a single mention by the systems (83.34%) the resulting numbers are very close. The average number of unique chemical name strings per abstract annotated manually for the test set was slightly higher (2.52) than the number of predicted unique compound names by returned by automated taggers (2.10).

### CHEMDNER silver standard corpus

Due to the considerable workload required for the construction of manually annotated corpora, some efforts have been made to construct automatically tagged text collections generated by different systems. Despite obvious limitations when relying on automated tagging, one advantage of this strategy is that they can generate very large datasets. When assuming that the automated tools have an acceptable performance, the combination of multiple systems can generate labels with an acceptable quality.

The *BioCreative metaserver *constituted a pioneering work in the integration, alignment and visualization of multiple automated predictions, including the annotation of gene/protein mentions and handling their character overlaps [49]. The use of silver standard corpora as training data was explored for the implementation of chunkers of biomedical text [50] and NER systems [51]. Usually the creation of silver standard corpora required a corpus harmonization in order to merge multiple predictions, in the simplest case by applying a voting scheme [13] together with various mention boundary reconciliation strategies (e.g. exact, nested, continuous similarity measure for mention alignments [13]). To help in the exploration of silver standard corpora usage for chemical entity recognition and explore alternative corpus construction strategies we have included the release the *CHEMDNER silver standard raw corpus*. The distribution of this corpus might allow the study of generalization strategies to a broader abstract collection. This corpus contains automatically generated chemical mention annotations generated by teams that participated in the BioCreative CHEMDNER task for a background collection of 17,000 PubMed abstracts. These abstracts corresponded to a random sample retrieved by a PubMed search carried out the 27th of August 2013 selecting records published during 2013 in English, with abstracts and links to full text papers, without any prior keyword or topic filtering. These articles were published in over 3,000 different journals. Originally this background set was added to the test set abstracts during the prediction phase of the CHEMDNER task to assure that teams did not have enough time to do any manual correction of their submissions, making sure that everything was done automatically. This set was also added to obtain predictions of abstracts that were not specifically pre-selected for chemistry. All automatic annotations distributed in the CHEMDNER silver corpus were in a *common format, enabling direct comparison and alignment of predictions*. This corpus contains only the crude annotations. By doing this we intend to promote that researchers explore their own cross comparison, mention alignment and consensus annotations strategies. A total of 8,359,524 automatic annotations by 105 runs were generated for these 17,000 abstracts. On average, the number of chemical mentions per abstract was of 4.39, almost half when compared to the chemistry-related test set abstracts. The number of predicted unique compound names per abstracts was 0.83 (compared to the 2.10 of the test set). These numbers partially reflect also the fact that in case of this random background set, on average only 52.80% of the abstracts did contain chemical mentions. When extrapolating these numbers to the entire PubMed database, of over currently 14,8 million records with abstracts, we would obtain over *12 million unique chemical names with more than 65 million mentions*. However these numbers have to be taken with care, because the background set corresponded to recent articles, while the PubMed database hosts a considerable number of older publications.

## Discussion and conclusions

**Figure 4:**
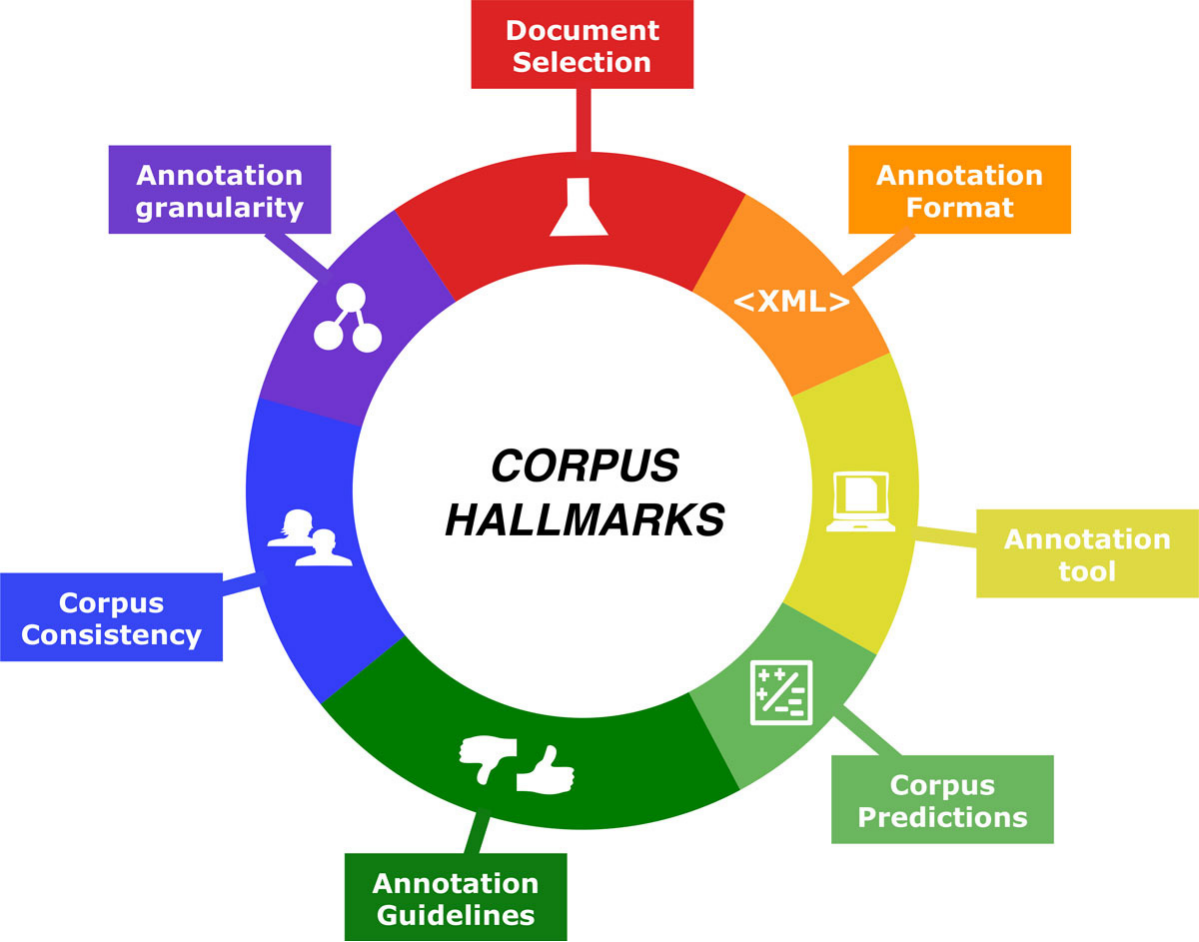
**The hallmarks of text corpus construction that were applied to the BioCreative CHEMDNER task**.

The CHEMDNER corpus is a publically available, manually annotated, machine-readable text corpus large enough to train chemical entity taggers. It is representative of modern chemical language (recent papers) for a range of central chemical disciplines. During the construction of this corpus, we have defined several *corpus hallmarks *that are key for the construction of manually annotated text corpora, not only for the chemical domain. These proposed hallmarks characterizing the CHEMDNER corpus are summarized in Figure 4. We consider it crucial to provide *minimal information *for each of these essential aspects of corpus construction. Prior to the construction of the CHEMDNER corpus, we encountered a range of problems with previous studies, related to corpus availability, lack of proper documentation, lack of document selection criteria, not enough information on annotation guidelines or problems with the corpus format. For the annotation of chemical entity mentions we believe that curators need to consider the entire abstract as context for manual annotation, beyond individual sentences. Chemical entity annotations should be done at the character level and not at the level of individual word tokens due to the intrinsic challenges of tokenizing chemical texts [33]. We think that the CHEMDNER corpus could be a valuable resource not only for entity recognition but also for the implementation of improved chemical text processing software (chemistry-tuned tokenization methods optimized for the correct identification of chemical entities) or to develop text categorization systems for triage of documents that do contain chemical mentions for manual curation. This corpus can potentially be used for the implementation of sub-domain specific chemical taggers tuned for more fine-grained chemistry disciplines. Through the examination of both manually annotated and automatically extracted chemical mentions, it should be possible to better understand the *chemical vocabulary *and define the *chemical space *of published articles. The CHEMDNER corpus and the taggers developed with it can be used to generate lexical resources, i.e. gazetteers, containing chemical entities: For example, previous studies showed that IUPAC names are poorly covered by existing chemical dictionaries [35]. The recognition of this type of chemical names can thus only be addressed either by machine learning and/or rule-based approaches that certainly benefit from the availability of manually labeled text like the CHEMDNER corpus. Considering the competitive performance of systems trained on the CHEMDNER corpus, we expect that these could be effective to generate pre-annotations that in turn can then be manually validated or corrected in a quick curation procedure. The CHEMDNER silver standard corpus can be interpreted as a sort of collaborative effort to annotate chemical entities. For this dataset there are still aspects that would benefit from further analysis, such as alternative harmonization strategies of the mentions or a comparative analysis on the performance of systems trained on silver-standard corpora versus gold standard corpora. The release of automatically extracted chemical mentions from the entire PubMed database (of systems trained the CHEMDNER corpus) would demonstrate how scalable those methods are and help in the curation of chemical data from PubMed. Moreover, determining ways to differentiate those mentions that are of practical relevance for curators still needs additional analysis, but some preliminary studies that took into account simply the position of the chemical names in the text and restricting the selection to certain sections of the abstract showed interesting outcomes [26]. The performance of these tools on other documents, including patents and full text articles could potentially highlight both the adaptability as well as challenges associated with each particular document type. For an enhanced version of the CHEMDNER corpus, aspects that could improve the impact of this resource include a more granular classification of the SACEM classes. With this respect, a simple ontology or hierarchical classification of chemical entity mention classes would be important. The underlying classes would have to be useful to improve automatic detection of entities and to facilitate the normalization of mention to either structures or chemical databases. Some mention classes can only be normalized using a dictionary based approach, others using name to structure software. Some of the current entities contained in the CHEMDNER dataset cannot be directly normalized without some more granular mention subtypes (e.g. in case of the SACEM class FAMILY). Well-specified, generally used workflows of the underlying normalization process of chemical entity mentions to structures/databases are currently missing. We also think that a more granular annotation strategy could help to improve the recognition of other entity mentions such as genes and proteins. In addition to a more detailed chemical mention classification, some annotations would benefit from a more granular labeling at the level of substrings, for instance in case of hybrid chemical mentions (e.g. chemical mentions that are formed by strings belonging to different SACEM classes like SYSTEMATIC and TRIVIAL). In the case of chemical mentions of the class MULTIPLE, which cover chemical entities that appear in form of separated or unconnected expressions (discontinuous) they are being annotated together in order to generate integrated forms. Improvement of this type of mention would require defining dependencies/relationships between the token spans. The CHEMDNER corpus currently is only concerned with chemistry-related information, missing annotation of linguistic aspects, syntactic and grammatical information. Adding this kind of information goes beyond the scope of this corpus, but could potentially be useful for other natural language processing tasks. Finally, the annotation of named entities, although a key step, is only the first task for the subsequent extraction of more practically useful information, such as chemical interactions. Annotation of a predefined set of relation types involving chemicals from the CHEMDNER corpus could help to promote research in the area of chemical relation extraction.

## Competing interests

The authors declare that they have no competing interests.

## Authors' contributions

MK was responsible for the task definition and coordinated the corpus annotation and result evaluation. FL and MV helped define the task, the annotations, and the evaluation of the results. OR and JO were responsible for refining the annotation guidelines and supervised the annotation quality and results. AV supervised the entire task setting. All authors revised the manuscript.

## Supplementary Material

Additional file 1 Click here for file

Additional file 2 Click here for file

## Declarations

We would like to acknowledge funding for this publication by the Innovative Medicines Initiative Joint Undertaking (IMI-eTOX, under Grant Agreement nr. 115002)

This article has been published as part of *Journal of Cheminformatics *Volume 7 Supplement 1, 2015: Text mining for chemistry and the CHEMDNER track. The full contents of the supplement are available online at http://www.jcheminf.com/supplements/7/S1.

## References

[B1] Vazquez M Krallinger M Leitner F Valencia A Text mining for drugs and chemical compounds: methods, tools and applications Molecular Informatics 2011 30 6-7 506 519 10.1002/minf.201100005 27467152

[B2] Krallinger M Valencia A Hirschman L Linking genes to literature: text mining, information extraction, and retrieval applications for biology Genome Biol 2008 9 Suppl 2 8 10.1186/gb-2008-9-s2-s8 18834499 PMC2559992

[B3] Leaman R Gonzalez G Banner: an executable survey of advances in biomedical named entity recognition Pacific Symposium on Biocomputing 2008 13 652 663 18229723

[B4] Gerner M Nenadic G Bergman CM Linnaeus: a species name identification system for biomedical literature BMC bioinformatics 2010 11 1 85 10.1186/1471-2105-11-85 20149233 PMC2836304

[B5] He M Wang Y Li W Ppi finder: a mining tool for human protein-protein interactions PloS one 2009 4 2 4554 10.1371/journal.pone.0004554 19234603 PMC2641004

[B6] Krallinger M Leitner F Valencia A Analysis of biological processes and diseases using text mining approaches Bioinformatics Methods in Clinical Research 2010 Humana Press 341 382 10.1007/978-1-60327-194-3_16 19957157

[B7] Krallinger M Izarzugaza JM Rodriguez-Penagos C Valencia A Extraction of human kinase mutations from literature, databases and genotyping studies BMC bioinformatics 2009 10 Suppl 8 1 19758464 10.1186/1471-2105-10-S8-S1 PMC2745582

[B8] Fontaine J-F Barbosa-Silva A Schaefer M Huska MR Muro EM Andrade-Navarro MA Medlineranker: flexible ranking of biomedical literature Nucleic acids research 2009 37 suppl 2 141 146 19429696 10.1093/nar/gkp353 PMC2703945

[B9] Leser U Hakenberg J What makes a gene name? named entity recognition in the biomedical literature Briefings in Bioinformatics 2005 6 4 357 369 10.1093/bib/6.4.357 16420734

[B10] Nadeau D Sekine S A survey of named entity recognition and classification Lingvisticae Investigationes 2007 30 1 3 26 10.1075/li.30.1.03nad

[B11] Krallinger M Leitner F Rabal O Vazquez M Oryazabal J Valencia A CHEMDNER: The drugs and chemical names extraction challenge J Cheminform 2015 7 Suppl 1 S1 10.1186/1758-2946-7-S1-S1 PMC4331685 25810766

[B12] Neves M Leser U A survey on annotation tools for the biomedical literature Briefings in bioinformatics 2012 084 23255168 10.1093/bib/bbs084

[B13] Rebholz-Schuhmann D Jimeno-Yepes AJ van Mulligen EM Kang N Kors JA Milward D Corbett PT Buyko E Tomanek K Beisswanger E The calbc silver standard corpus for biomedical named entities-a study in harmonizing the contributions from four independent named entity taggers LREC 2010 10.1142/s0219720010004562 20183881

[B14] Kim J-D Ohta T Tateisi Y Tsujii J Genia corpus-semantically annotated corpus for bio-textmining Bioinformatics 2003 19 suppl 1 180 182 10.1093/bioinformatics/btg1023 12855455

[B15] Lipscomb CE Medical subject headings (mesh) Bulletin of the Medical Library Association 2000 88 3 265 10928714 PMC35238

[B16] Bada M Eckert M Evans D Garcia K Shipley K Sitnikov D Baumgartner WA Cohen KB Verspoor K Blake JA Concept annotation in the craft corpus BMC bioinformatics 2012 13 1 161 10.1186/1471-2105-13-161 22776079 PMC3476437

[B17] Degtyarenko K de Matos P Ennis M Hastings J Zbinden M McNaught A Alcántara R Darsow M Guedj M Ashburner M Chebi: a database and ontology for chemical entities of biological interest Nucleic acids research 2008 36 suppl 1 344 350 17932057 10.1093/nar/gkm791 PMC2238832

[B18] Van Mulligen EM Fourrier-Reglat A Gurwitz D Molokhia M Nieto A Trifiro G Kors JA Furlong LI The eu-adr corpus: Annotated drugs, diseases, targets, and their relationships Journal of biomedical informatics 2012 45 5 879 884 10.1016/j.jbi.2012.04.004 22554700

[B19] Wishart DS Knox C Guo AC Shrivastava S Hassanali M Stothard P Chang Z Woolsey J Drugbank: a comprehensive resource for in silico drug discovery and exploration Nucleic acids research 2006 34 suppl 1 668 672 16381955 10.1093/nar/gkj067 PMC1347430

[B20] Herrero-Zazo M Segura-Bedmar I Martínez P Declerck T The ddi corpus: An annotated corpus with pharmacological substances and drug-drug interactions Journal of biomedical informatics 2013 46 5 914 920 10.1016/j.jbi.2013.07.011 23906817

[B21] Rindflesch TC Tanabe L Weinstein JN Hunter L Edgar: extraction of drugs, genes and relations from the biomedical literature Pacific Symposium on Biocomputing. Pacific Symposium on Biocomputing. NIH Public Access 2000 517 10902199 10.1142/9789814447331_0049 PMC2709525

[B22] Nobata C Dobson PD Iqbal SA Mendes P Tsujii J Kell DB Ananiadou S Mining metabolites: extracting the yeast metabolome from the literature Metabolomics 2011 7 1 94 101 10.1007/s11306-010-0251-6 21687783 PMC3111869

[B23] Schlaf A Bobach C Irmer M Chair, NCC, Choukri K, Declerck T, Loftsson H, Maegaard B, Mariani J, Moreno A, Odijk J, Piperidis S Creating a gold standard corpus for the extraction of chemistry-disease relations from patent texts Proceedings of the Ninth International Conference on Language Resources and Evaluation (LREC'14) 2014 European Language Resources Association (ELRA), Reykjavik, Iceland

[B24] Wilbur WJ Hazard GF Jr Divita G Mork JG Aronson AR Browne AC Analysis of biomedical text for chemical names: a comparison of three methods Proceedings of the AMIA Symposium, American Medical Informatics Association 1999 176 10566344 PMC2232672

[B25] Wren JD A scalable machine-learning approach to recognize chemical names within large text databases BMC bioinformatics 2006 7 Suppl 2 3 10.1186/1471-2105-7-S2-S3 17118146 PMC1683569

[B26] Zhang JD Geer LY Bolton E Bryant SH Automated annotation of chemical names in the literature with tunable accuracy J Cheminformatics 2011 3 52 10.1186/1758-2946-3-52 22107874 PMC3281788

[B27] Narayanaswamy M Ravikumar K Vijay-Shanker K Ay-shanker KV A biological named entity recognizer Pac Symp Biocomput 2003 427 12603047 10.1142/9789812776303_0040

[B28] Hawizy L Jessop DM Adams N Murray-Rust P Chemicaltagger: A tool for semantic text-mining in chemistry Journal of cheminformatics 2011 3 1 17 10.1186/1758-2946-3-17 21575201 PMC3117806

[B29] Standard, C.P.G http://chebi.cvs.sourceforge.net/viewvc/chebi/chapati/patentsGoldStandard

[B30] Tiago G Catia P Bastos Hugo P Chemical entity recognition and resolution to chebi ISRN Bioinformatics 2012 2012 10.5402/2012/619427 PMC4393067 25937941

[B31] Akhondi SA Klenner AG Tyrchan C Manchala AK Boppana K Lowe D Zimmermann M Jagarlapudi SA Sayle R Kors JA Annotated chemical patent corpus: A gold standard for text mining PloS one 2014 9 9 107477 10.1371/journal.pone.0107477 25268232 PMC4182036

[B32] Rupp C Copestake A Teufel S Waldron B Flexible interfaces in the application of language technology to an escience corpus Proceedings of the UK e-Science Programme All Hands Meeting. Citeseer 2006

[B33] Corbett P Batchelor C Teufel S Annotation of chemical named entities Proceedings of the Workshop on BioNLP 2007: Biological, Translational, and Clinical Language Processing. Association for Computational Linguistics 2007 57 64

[B34] Corbett P Copestake A Cascaded classifiers for confidence-based chemical named entity recognition BMC bioinformatics 2008 9 Suppl 11 4 10.1186/1471-2105-9-S11-S4 19025690 PMC2586753

[B35] Kolárik C Klinger R Friedrich CM Hofmann-Apitius M Fluck J Chemical names: terminological resources and corpora annotation Workshop on Building and Evaluating Resources for Biomedical Text Mining (6th Edition of the Language Resources and Evaluation Conference) 2008

[B36] Tamames J Valencia A The success (or not) of hugo nomenclature Genome biology 2006 7 5 402 10.1186/gb-2006-7-5-402 16707004 PMC1779514

[B37] Arighi CN Wu CH Cohen KB Hirschman L Krallinger M Valencia A Lu Z Wilbur JW Wiegers TC Biocreative-iv virtual issue Database 2014 2014 039 10.1093/database/bau039 PMC4030502 24852177

[B38] Yeh A Morgan A Colosimo M Hirschman L Biocreative task 1a: gene mention finding evaluation BMC bioinformatics 2005 6 Suppl 1 2 10.1186/1471-2105-6-S1-S2 15960832 PMC1869012

[B39] Smith L Tanabe LK Ando RJ Kuo CJ Chung IF Hsu CN Lin YS Klinger R Friedrich CM Ganchev K Torii M Liu H Haddow B Struble CA Povinelli RJ Vlachos A Baumgartner WA Hunter L Carpenter B Tsai RT Dai HJ Liu F Chen Y Sun C Katrenko S Adriaans P Blaschke C Torres R Neves M Nakov P Divoli A Mana-Lopez M Mata J Wilbur WJ Overview of BioCreative II gene mention recognition Genome Biol 2008 9 Suppl 2 2 10.1186/gb-2008-9-s2-s2 18834493 PMC2559986

[B40] Hirschman L Colosimo M Morgan A Yeh A Overview of biocreative task 1b: normalized gene lists BMC bioinformatics 2005 6 Suppl 1 11 10.1186/1471-2105-6-S1-S11 15960823 PMC1869004

[B41] corpus, C http://www.biocreative.org/resources/biocreative-iv/chemdner-corpus

[B42] Salgado D Krallinger M Depaule M Drula E Tendulkar AV Leitner F Valencia A Marcelle C Myminer: a web application for computer-assisted biocuration and text annotation Bioinformatics 2012 28 17 2285 2287 10.1093/bioinformatics/bts435 22789588

[B43] tool, A http://annotateit.org

[B44] Ide N Romary L Representing linguistic corpora and their annotations Proceedings of the Fifth Language Resources and Evaluation Conference (LREC), Genoa, Italy 2006

[B45] Comeau DC Doğan RI Ciccarese P Cohen KB Krallinger M Leitner F Lu Z Peng Y Rinaldi F Torii M Bioc: a minimalist approach to interoperability for biomedical text processing Database 2013 2013 064 10.1093/database/bat064 PMC3889917 24048470

[B46] Smith L Tanabe LK Ando RJ Kuo C-J Chung I-F Hsu C-N Lin Y-S Klinger R Friedrich CM Ganchev K Overview of biocreative ii gene mention recognition Genome biology 2008 9 Suppl 2 2 10.1186/gb-2008-9-s2-s2 PMC2559986 18834493

[B47] Leitner F Mardis SA Krallinger M Cesareni G Hirschman LA Valencia A An overview of biocreative ii. 5 Computational Biology and Bioinformatics, IEEE/ACM Transactions on 2010 7 3 385 399 10.1109/tcbb.2010.61 20704011

[B48] 2, B.I.-C.P.V http://www.biocreative.org/resources/publications/chemdner-proceed-publications

[B49] Leitner F Krallinger M Rodriguez-Penagos C Hakenberg J Plake C Kuo C-J Hsu C-N Tsai R Hung H-C Lau WW Introducing meta-services for biomedical information extraction Genome Biol 2008 9 Suppl 2 6 10.1186/gb-2008-9-s2-s6 18834497 PMC2559990

[B50] Kang N van Mulligen EM Kors JA Training text chunkers on a silver standard corpus: can silver replace gold? BMC bioinformatics 2012 13 1 17 10.1186/1471-2105-13-17 22289351 PMC3280170

[B51] Rebholz-Schuhmann D Yepes AJ Li C Kafkas S Lewin I Kang N Corbett P Milward D Buyko E Beisswanger E Assessment of ner solutions against the first and second calbc silver standard corpus Journal of biomedical semantics 2011 2 5 1 12 22166494 10.1186/2041-1480-2-S5-S11 PMC3239301

