# Supplementary material for: The CHEMDNER corpus of chemicals and drugs and its annotation principles
Source: J Cheminform. 2015 Jan 19;7(Suppl 1):S2. doi: 10.1186/1758-2946-7-S1-S2 (PMC4331692; doi:10.1186/1758-2946-7-S1-S2)
Supplement: Additional file 1 [file 1758-2946-7-S1-S2-S1.pdf]

A. J Cheminform CHEMDNER special issue

A.1. CHEMDNER challenge overview paper with summary tables of systems performance, methods, features and used resources:

- Martin Krallinger, Florian Leitner, Obdulia Rabal, Miguel Vazquez, Julen Oyarzabal, Alfonso Valencia, A. CHEMDNER: The drugs and chemical names extraction challenge. J Cheminform

A.2. Participating teams detailed system description/update papers (selected after peer review process):

Team 173 : Robert Leaman, Chih-Hsuan Wei and Zhiyong Lu. tmChem: a high performance approach for chemical named entity recognition and normalization. J Cheminform
Team 231: Yanan Lu, Xiaoyuan Yao, Xiaomei Wei, Donghong Ji and Xiaohui Liang. CHEMDNER System with Mixed Conditional Random Fields and Multi-scale Word Clustering. J Cheminform
Team 179: Daniel M Lowe and Roger A Sayle. LeadMine: A grammar and dictionary driven approach to entity recognition. J Cheminform
Team 184: Riza Batista-Navarro, Rafal Rak and Sophia Ananiadou. Optimising chemical named entity recognition with pre-processing analytics, knowledge-rich features and heuristics. J Cheminform
Team 197: David Campos, Sérgio Matos and José L Oliveira. A document processing pipeline for annotating chemical entities in scientific documents. J Cheminform
Team 192: Buzhou Tang Dr, Yudong Feng Dr, Xiaolong Wang Prof, Yonghui Wu Dr, Yaoyun Zhang Dr, Min Jiang Ms, Jingqi Wang Ms and Hua Xu Prof. A Comparison of Conditional Random Fields and Structured Support Vector Machines for Chemical Entity Recognition in Biomedical Literature. J Cheminform
Team 233: Tsendsuren Munkhdalai, Meijing Li, Khuyagbaatar Batsuren, Hyeon Ah Park, Nak Hyeon Choi and Keun Ho Ryu. Incorporating domain knowledge in chemical and biomedical named entity recognition with word representations. J Cheminform
Team 222: Saber A Akhondi, Kristina M Hettne, Eelke van der Horst, Erik M van Mulligen and Jan A Kors. Recognition of chemical entities: combining dictionary-based and grammar-based approaches. J Cheminform
Team 259: Shuo Xu, Xin An, Lijun Zhu, Yunliang Zhang and Haodong Zhang. A CRF-Based System for Recognizing Chemical Entity Mentions (CEMs) in Biomedical Literature. J Cheminform
Team 219: Madian Khabsa and C. Lee Giles. Chemical Entity Extraction using CRF and an Ensemble of Extractors. J Cheminform
Team 196: Andre Lamurias, João D Ferreira and Francisco M Couto. Improving chemical entity recognition through h-index based semantic similarity. J Cheminform
Team 265: Hong-Jie Dai, Johnny Chi-Yang Wu, Po-Ting Lai, Yung-Chun Chang and Richard Tzong-Han Tsai . Enhancing of Chemical Compound and Drug Name Recognition Using Representative Tag Scheme and Fine-grained Tokenization. J Cheminform
Team 191: Ana Usié, Joaquim Cruz, Jorge Comas, Francesc Solsona and Rui Alves. CheNER: A tool for the differential identification of several classes of chemical entities. J Cheminform

B. Proceedings of the Fourth BioCreative Challenge Evaluation Workshop vol. 2.

Collection of short technical systems description papers by participating teams submitted for the CHEMDNER evaluation workshop proceedings.
Available at:
http://www.biocreative.org/resources/publications/chemdner-proceed-publications

C. Short system descriptions provided by participating teams (sorted by CEM task f-scores)

Team 173

Authors: Zhiyong Lu[1] and Robert Leaman[1]
Affiliation: [1] National Center for Biotechnology Information (NCBI), National Institutes of Health, Bethesda, USA
e-mail: ZL: zhiyong.lu@nih.gov; RL: robert.leaman@nih.gov

SHORT SYSTEMS DESCRIPTION
The NCBI entry to the CHEMDNER task, tmChem, combines two conditional random field (CRF) models and multiple post-processing steps. The first model is an adaptation of BANNER [1], built on the MALLET toolkit (http://mallet.cs.umass.edu/), using a fine tokenization which splits tokens at whitespace, punctuation, digits, and lowercase letters followed by an upper case letter. The feature set includes a wide array of features typically used for NER, the output of ChemSpot [2] – which provides functionality similar to a lexicon but with increased flexibility – and multiple pattern features to recognize chemical elements, formulas and amino acids. The second model uses the CRF++ library (https://code.google.com/p/crfpp/), and is repurposed from the tmVar system for locating genetic variants [3]. This model uses an even finer tokenization than Model 1 by also separating digits, lower and upper case letters into separate tokens. Model 2 also employs a wide variety of typical NER features, features to recognize chemiCEM elements and also many morphemes semantically meaningful in chemical names (e.g. "tri-" and "-ate"). Our models employ post processing steps for several purposes, including resolving abbreviations using Ab3P [4], improving consistency, balancing parentheses, and also identifying chemical identifiers by matching to a dictionary extracted from the CTD database (http://ctdbase.org/). We experimented with model combinations, marginal probabilities to provide high recall results, and also a method to convert our CEM results to the CDI task. Our highest performance on the test set for the CDI task was an f-measure of 87.52%. Our highest performance on the test set for the CEM task was an f-measure of 87.39%, the highest in the task, and our highest recall was 92.12%.

References
1. Leaman, R., Gonzalez, G.: BANNER: an executable survey of advances in biomedical named entity recognition. Pac. Symp. Biocomput. 652-663 (2008)
2. Rocktaschel, T., Weidlich, M., Leser, U.: ChemSpot: A Hybrid System for Chemical Named Entity Recognition. Bioinformatics 28, 1633-1640 (2012)

3. Wei, C.-H., Harris, B.R., Kao, H.-Y., Lu, Z.: tmVar: A text mining approach for extracting sequence variants in biomedical literature. Bioinformatics 29, 1433-1439 (2013)
4. Sohn, S., Comeau, D.C., Kim, W., Wilbur, W.J.: Abbreviation definition identification based on automatic precision estimates. BMC Bioinformatics 9, 402 (2008)

Team 231

Authors: Yanan Lu[1]* and Donghong Ji [1§]
Affiliation: [1]Natual Language Processing Lab, Wuhan University, Luojia Hill, Wuchang District, Wuhan 430072, Hubei, China
e-mail: YL: luyanan@whu.edu.cn; DJ: dhji@whu.edu.cn
*These authors contributed equally to this work
[§]Corresponding author

SHORT SYSTEMS DESCRIPTION

WHU-BioNLP CHEMDNER System with Mixed Conditional Random Fields and Word Clustering

We considered the chemical compound and drug name recognition as a sequence labeling problems, and conditional random fields [2] is the classic method to solve this problem. We use CRFs to as the main frame to design CHEMDNER system for recognizing the chemical compound and drug name in the paper, the main frame of the system is as Figure 1. We will introduce the system and the methods in four aspects, preprocessing, word clustering, mixed CRFs models and post processing.

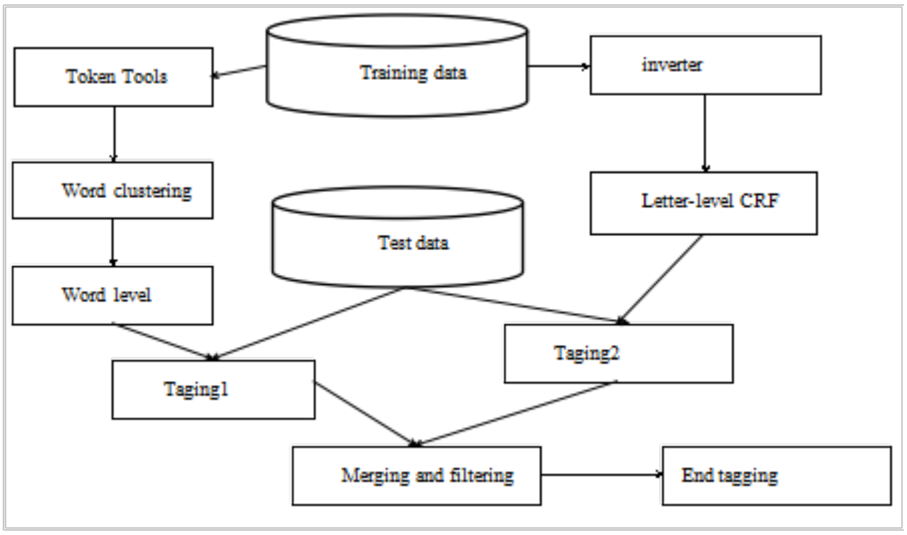

Figure 1 The frame of CHEMDNER System

Preprocessing

We propose two methods to preprocess. First we construct the tokenizer with white space and the symbols in Figure 1 to segment the corpus, which can reduce the sparse feature in the BioCreative IV task.

~ · ! @ # $ % ^ & * - = _ + ¯ ( ) [ ] { } ; ' : " , . / <> × ＞ ＜ ⩽ ⩾ ↑ ↓ ← → ·
′ ° ∼ ≈ ? Δ ÷ ≠ | ‘ ’ “ ” § ￡ € \ 0 1 2 3 4 5 6 7 8

Figure 2 Symbols included in tokenizer

Furthermore, previous studies show that the F-measure of the recognition can be improved in the letter level and with the training from the right to the left (**Error! Reference source not found.**). In this study, we also constructed a CRF model in the letter level, with the training from the right to the left.

Word clustering

Our system uses the Brown Cluster, created from a hierarchical word clustering algorithm, and we downloaded about 74,000 articles including titles and abstracts from the PubMed database, and segmented them with the tokenizer for word clustering. We used the Brown clustering algorithm to acquire 1,000 clusters, and assigned each cluster a binary representation based on Huffman coding. Table 2 shows some example words and their binary representations. Intuitively, the more similar the prefix of the word's Huffman codings, the more similar the words.

Training and prediction

When training the corpus, we used the label set {B, M, E, S, B_NER, M_NER, E_NER, S_NER}. B stands for the beginning of a normal sequence, M for the middle of the normal sequence, E for the end of the normal sequence, S for the single-token normal sequence. B_NER stands for the beginning of an entity sequence and so on. The reason why we didn't use the label set {B, I, O} in the system is that the label set in the system exhibited a better distinguishability in this task. We used three methods shown in Table1 to train the model with 7,000 training data and 3,000 test data, and merged the two tagging results based on the following strategy: when the two results have the same left offset or right offset, we choose the one with the higher confidence if its confidence higher than the other by 0.7; otherwise we keep the longer one and delete the shorter one. After merging the results, we filtered the entity if its confidence is lower than 0.00001. Finally, we refined the result with the strategies listed in Table 1. Take the row 1 as an example, it means that when the count of '(' is higher than that of ')' by 1 in the entity, and if to the right of the entity

Table 2: The strategy to refine the result

Count(symbol) in entity	Symbol next to entity	Change offset
(=) + 1	Right is)	Right + 1
(=) - 1	Left is (	Left – 1
[=] + 1	Right is]	Right + 1
[=] - 1	Left is [	Left - 1
{ = }+ 1	Right is }	Right + 1
{ = } - 1	Left is {	Left - 1

Results and discussion

Our system trained on 7000 annotated PubMed corpus and tested on 3000 PubMed corpus. Both the training corpus and testing corpus were provided by BioCreative IV, which also provided the evaluation method and the evaluation tool. And the result for CDI task is shown in the Table 3, and result for CEM shown in the table 4. In the BioCreative IV evaluation, our system achieved the highest F-score of 88.20 % in CDI task and achieved the second F-score of 87.11, which lower 0.28% than the highest F-sore of 87.39%.

Table 3: CDI result by our system

System	Precision	Recall	F-score
Letter	0.90706	0.85121	0.87825
Word	0.90221	0.74536	0.81632
Letter+invert	0.90766	0.85157	0.87872
Word+Brown	0.89322	0.78393	0.83501
Merge system	0.87018	0.89408	0.88197

Table 4: CEM result by our system

System	Precision	Recall	F-score
Letter	0.91965	0.80450	0.85823
Word	0.91774	0.70632	0.79827
Letter+invert	0.91992	0.80482	0.85853
Word+Brown	0.91069	0.74530	0.81973
Merge system	0.89105	0.85200	0.87109

Analysis and conclusion

From the table 3 and table 4, we can obtain the conclusion the inverting the corpus improved the performance a litter, and word clustering improved the recall rate of the system. Thus the word clustering improved the F-score of 1.87% in the CDI task and 2.15% in the CEM task. And we improved the all F-score of 0.37% in the CDI task and the mixed the model 1.29% in the CEM task.

Acknowledgements

Thanks to High performance computing center of computer school, University, for supporting us that we can do large-scale corpus experiment. The work is funded by the following projects: Natural Science Foundation of China No.61133012 and No.61202304.

Team 179

Authors: Daniel M. Lowe[*1] and Roger A. Sayle[1]

Affiliation: [1]NextMove Software Ltd, Innovation Centre, Unit 23, Science Park, Milton Road, Cambridge, United Kingdom

e-mail: DML: daniel@nextmovesoftware.com; RAS: roger@nextmovesoftware.com

SHORT SYSTEMS DESCRIPTION

LeadMine is a system for recognizing entities, especially chemical entities, using a combination of expertly curated grammars and dictionaries, as well as dictionaries automatically derived from public resources. The identified entities are directly related to a given grammar or dictionary, which allows the type of an entity to be known and, if an entity is misannotated, indicates which resource should be corrected. As recognition is driven by what is expected the approach can correct spelling errors, which can assist

with looking up an entity in a database or converting a chemical name to a structure. To achieve state of the art performance (87.6% F_1 -score on the CEM task) the system uses a high precision dictionary derived from PubChem in combination with entity boundary modification and abbreviation detection post-processing steps.

Team 184

Authors: Riza Theresa Batista-Navarro[1] and Rafal Rak[1]
Affiliation: [1] National Centre for Text Mining, Manchester Institute of Biotechnology 131 Princess St., Manchester,United Kingdom M1 7DN
e-mail: RTBN: riza.batista-navarro@cs.man.ac.uk; RR: rafal.rak@manchester.ac.uk

SHORT SYSTEMS DESCRIPTION

For both CDI and CEM tasks, the challenge was cast as a sequence labelling problem which was addressed using the conditional random fields (CRF) algorithm (Lafferty et al., 2001). The documents were pre-processed with the LingPipe MEDLINE sentence model (http://alias-i.com/lingpipe), the tokeniser of OSCAR4 (Jessop et al., 2011) and the GENIA part-of-speech and chunk tagger (Tsuruoka et al., 2005). With the utilisation of the NERsuite package (http://nersuite.nlplab.org), a statistical model was trained based on a rich feature set that was customised to capture various chemical characteristics. Engineered features included matches within chemical dictionaries, the occurrence of chemical affixes, the number of chemical basic segments, and matches against a list of chemical element symbols. The used dictionaries were ChEBI (de Matos et al., 2010), DrugBank (Knox et al., 2011), CTD (Davis et al., 2012), PubChem Compound (Bolton et al., 2008) and the Joint Chemical Dictionary (Hettne et al., 2009). Furthermore, post-processing heuristics were incorporated into the method to capture missed abbreviations and to re-label tokens initially recognised as non-chemical but likely to be constituents of chemical names. Evaluation on the provided data set reveals that the incorporation of additional features and post-processing heuristics improved the performance for both CDI and CEM, compared to employing only a model trained on the basic features typically used for NER.

Team 198

Authors: Torsten Huber[1] and Tim Rocktäschel[2]
Affiliation: [1] Humboldt-Universit ̈at zu Berlin,Knowledge Management in Bioinformatics, Unter den Linden 6, Berlin, 10099, Germany. [2] Department of Computer Science, University College London, United Kingdom
e-mail: TH: thuber@informatik.hu-berlin.de; TR: ucabroc@ucl.ac.uk

SHORT SYSTEMS DESCRIPTION

Different naming conventions of chemicals (e.g. trivial, systematic or brand names) and their incoherent usage lead to morphologically diverse mentions of chemicals in text. Building on our chemical Named Entity Recognition tool ChemSpot [1], we address this diversity in [2] by training a second-order linear-chain conditional random field (CRF) with features extracted from the output of subroutines of ChemSpot and OSCAR4 [3], as well as containment of token n-grams in various chemical dictionaries and ontologies. Compared to our previous system [4], we added as features predictions of OSCAR4, dictionaries generated from PHARE, Drugs@FDA and WHO ATC, as well as token skip-grams and word stems. The resulting meta model captures individual confidences and biases of these resources with respect to the task at hand.

Our ablation study revealed that features derived from OSCAR4 and dictionaries in Jochem [5], particularly MeSH, have the highest impact on the quality of predictions on the development set (see [2] for more details). Compared to a configuration that solely employs standard features as reported in the Named Entity Recognition literature, using these domain-specific features in addition increases the performance on the test set substantially: From 55.11% to 84.17% F_1 measure. The best results were obtained when working with all feature classes but switching off stemming (86.47% F_1 measure).
For the chemical document indexing subtask, we generate a ranked list of chemicals by calculating TF-IDF scores for mentions predicted by the CRF, yielding an F_1 measure of 87.90%.

References
[1] T. Rocktäschel, M. Weidlich, and U. Leser, "ChemSpot: A Hybrid System for Chemical Named Entity Recognition," Bioinformatics, vol. 28, no. 12, pp. 1633–1640, 2012.
[2] T. Huber, T. Rocktäschel, M. Weidlich, P. Thomas, and U. Leser, "Extended Feature Set for Chemical Named Entity Recognition and Indexing," BioCreative Challenge Evaluation Workshop vol. 2, 88, 2013.
[3] D.M. Jessop, S.E. Adams, E.L. Willighagen, L. Hawizy, and P.Murray-Rust, "OSCAR4: a flexible architecture for chemical text-mining," Journal of cheminformatics, vol. 3, no. 1, p. 41, 2011.
[4] T. Rocktäschel, T. Huber, M. Weidlich, and U. Leser, "WBI-NER: The impact of domain-specific features on the performance of identifying and classifying mentions of drugs." In Proc. of the 7th International Workshop on Semantic Evaluation (SemEval 2013). 2013.
[5] K.M. Hettne, R.H. Stierum, M.J. Schuemie, P.J. Hendriksen, B.J. Schijvenaars, E.M. Mulligen, J. Kleinjans, J.A. Kors. "A dictionary to identify small molecules and drugs in free text." Bioinformatics, 25(22):2983-2991, 2009.

Team 197
Authors: Sérgio Matos[1] and David Campos[1]
Affiliation: [1] IEETA/DETI, University of Aveiro, Campus Universitrio de Santiago, 3810-193 Aveiro, Portugal
e-mail: SM: aleixomatos@ua.pt; DC: david.campos@bmd-software.com

SHORT SYSTEMS DESCRIPTION
We applied a supervised machine-learning approach, using the Conditional Random Fields (CRFs) [1] implementation provided by MALLET [2]. The method applied for this work was developed on top of two frameworks: Gimli [3] was used for feature extraction and to train the machine learning (ML) models, and Neji [4] was used for performing NLP, for pre- and post-processing tasks and as the framework for multi-threaded document annotation.
We defined a complex set of features to properly represent these entities, including token and NLP derived features (lemma, POS, chunk tags, and dependency parsing features), orthographic features (number of digits and capitalized, symbols and Greek letters), morphological features (suffixes and prefixes, char n-grams of 2, 3 and 4 characters and word shape) and domain knowledge features derived from dictionary matching. We used conjunctions to encode local context, as this gave better results than using windows. To encode the domain knowledge, we performed preliminary tests using

dictionaries compiled from various sources, and finally created a dictionary with terms Jochem [5], ChEBI [6] and CTD [7].
For the final system, we combined two CRF models of orders 1 and 2. A simple algorithm was used to harmonize annotations provided by these two models. Namely, when two annotations from the different models intersect, the harmonization algorithm considers the confidence scores provided by each model and selects the intersecting annotations with the highest scores. If an annotation does not intersect with others, it is directly added to the final list of annotations. Additionally, we applied two post-processing modules in order to solve some errors generated by the CRF model. First, to perform parentheses correction, the number of parentheses on each annotation is verified and the annotation is removed if this is an odd number, as this usually indicates a mistake by the ML model. We also applied abbreviation resolution to add missing long or short forms, if the corresponding form was annotated, and to expand the annotation boundaries using the result of the abbreviation extraction tool.
Ranking was derived from the confidence scores provided by the CRF models, a value between 0 and 1 that reflects the certainty of the model generating each annotation. In that way, raking simply orders the annotations in descending order of scores. In the case of the CDI task, an additional filtering step was applied, to remove repeated annotations with the same case-insensitive text.

References
[1] Lafferty, J., McCallum, A., Pereira, F.: Conditional random fields: Probabilistic models for segmenting and labeling sequence data (2001)
[2] McCallum, A.K.: MALLET: A Machine Learning for Language Toolkit. http://mallet.cs.umass.edu
[3] Campos, D., Matos, S., Oliveira, J.L.: Gimli: open source and high-performance biomedical name recognition. BMC bioinformatics 14(1), 54 (2013)
[4] Campos, D., Matos, S., Oliveira, J.L.: A modular framework for biomedical concept recognition. BMC bioinformatics 14(281) (2013)
[5] Hettne, K.M., Stierum, R.H., Schuemie, M.J., Hendriksen, P.J.M., Schijvenaars, B.J.a., Mulligen, E.M.v., Kleinjans, J., Kors, J.a.: A dictionary to identify small molecules and drugs in free text. Bioinformatics (Oxford, England) 25(22), 2983–2991 (2009)
[6] Degtyarenko, K., De Matos, P., Ennis, M., Hastings, J., Zbinden, M., McNaught, A., Alc antara, R., Darsow, M., Guedj, M., Ashburner, M.: ChEBI: a database and ontology for chemical entities of biological interest. Nucleic acids research 36(suppl 1), 344–350 (2008)
[7] Davis, A.P., Murphy, C.G., Saraceni-Richards, C.A., Rosenstein, M.C., Wiegers, T.C., Mattingly, C.J.: Comparative Toxicogenomics Database: a knowledgebase and discovery tool for chemical-gene-disease networks. Nucleic acids research 37(Database issue), 786–92 (2009)

Team 192
Authors: Buzhou Tang [1,2] and Hua Xu[2]
Affiliation: [1] Department of Computer Science, Harbin Institute of Technology Shenzhen Graduate School Shenzhen, GuangDong, China. [2] School of Biomedical Informatics, The University of Texas Health Science Center at Houston, Houston, Texas, USA
e-mail: BZ: tangbuzhou@gmail.com; HX: Hua.xu@uth.tmc.edu

SHORT SYSTEMS DESCRIPTION

A machine learning-based system was proposed for chemical entity mention recognition (CEM). The system first used a rule-based module for sentence boundary detection and tokenization, and then built machine learning classifiers based on conditional random fields (CRF) and structured support vector machines (SSVM) on the training and development sets respectively. The chemical entities were represented by BIO tags, where “B”, “I” and “O” denote the beginning, inside, and outside of an entity respectively. The features used in the classifiers included orthographic information, morphological information, bag-of-word, part-of-speech, chemical lexicons, and three types of word representation related features (i.e., clustering-based word representation, distributional word representation, and distributed word representation). The SSVM-based classifier achieved an F-measure of 85.20% on the test set, which is better than the CRF-based classifier (85.15%).

Team 233

Authors: Tsendsuren Munkhdalai[1] and Keun Ho Ryu[1+]
Affiliation: [1] Database/Bioinformatics Laboratory, School of Electrical & Computer Engineering, Chungbuk National University, Cheongju, South Korea
e-mail: TM: tsendeemts@dblab.chungbuk.ac.kr; KHR: khryu@dblab.cbnu.ac.kr
[+]Corresponding Author

SHORT SYSTEMS DESCRIPTION

BANNER-CHEMDNER: Incorporating domain knowledge in chemical and biomedical named entity recognition

We proposed a semi-supervised learning method that exploits unlabeled data efficiently in order to incorporate the domain knowledge into named entity recognition model to leverage the overall system performance. The proposed method includes natural language processing tasks for text preprocessing, learns the word representation features from a large amount of text data for feature extraction and applies the CRF for token classification. Our method does not rely on any lexicon and any dictionary other than the free text in the domain in order to keep the system applicable to the other NER tasks in bio-text data, even though the usage of such resources is reported to be considerable in boosting the system performance.

Brown cluster labels [1] and word vector classes, both induced by learning a large amount of unlabeled text data are extracted as generally applicable word representation features in addition to the word, the word character n-gram and the character n-gram, and the traditional orthographic information (baseline features). K-means clustering was applied to word vector models to drive the word vector classes. We evaluated two different tokenization strategies, a simple white space tokenizer and BANNER simple tokenizer [2] on word representation feature extraction. Surprisingly, very basic whitespace tokenizer performed better.

For the unlabeled data, we collected around 1.4 million PubMed abstracts and full text articles of the whole PMC database available at the time (over 2 million documents). After preprocessing, we derived two different text corpora, PubMed abstract corpus consisting of 1,136,085 vocabularies for induction of Brown clustering models and a merged corpus of the both resources with 4,359,932 vocabularies for learning of word vector models. The combination of the word representation features and word lemmas provided by Biolemmatizer [3] boosted the system performance significantly.

We extended BANNER, a biomedical named entity recognition system with the proposed method. This yields an integrated system that can be applied for chemical and

drug NER or biomedical NER. We called our branch of the BANNER system as BANNER-CHEMDNER, which is scalable over millions of documents processing ~530 docs/min, configurable via XML, and pluggable in other systems thanking to BANNER UIMA interface.
BANNER-CHEMDNER achieves 85.679% and 86.465% of F-measures on the testing set of CHEMDNER CEM and CDI subtasks, and 87.043% of F-measure on the official testing set of BioCreative 2 gene mention task, showing a remarkable performance on both chemical and biomedical NER. In fact, the result reported above outperforms our official entries for CHEMDNER CEM and CDI subtasks by is 0.925% and 0.725% of F-measure, and is the second best performance among the BioCreative 2 gene mention task.

References
[1] Robert L, Graciela G: BANNER: AN EXECUTABLE SURVEY OF ADVANCES IN BIOMEDICAL NAMED ENTITY RECOGNITION. In Proceedings of Pacific Symposium on Biocomputing, 2008: 652-663.
[2] Peter FB, Peter VD, Robert LM, Vincent JDP, Jenifer CL: Class-Based n-gram Models of Natural Language. Computational Linguistics 1992, 18:467-479
[3] Haibin L, Tom C, William AB, Karin V: BioLemmatizer: a lemmatization tool for morphological processing of biomedical text. Journal of Biomedical Semantic 2012, 3:3

Team 185
Authors: S. V. Ramanan[1] and P. Senthil Nathan[1]
Affiliation: [1] RelAgent Pvt Ltd, #14, IIT Madras Research Park, Taramani, Chennai 600113, India
e-mail: SVR: ramanan@relagent.com; PSN: senthil@relagent.com

SHORT SYSTEMS DESCRIPTION
Adapting Cocoa, a multi-class entity detector, for the CHEMDNER task of BioCreative IV
Cocoa is an existing multi-class entity detection system for the biomedical domain. The linguistic system uses morphological information as well as small dictionaries for detecting chemical entities. We made minor changes to the system for the CHEMDNER task by: (a) trimming entities and excluding generic entity terms, both irrelevant to core name detection as defined in the task and (b) adding dictionary entries and (c) handling unusual entity mentions. The final performance in both tasks was ~5% lower than the best performer. We anticipate that adding a ML-based post-module will substantially improve system performance, based on the methodology of other teams in the task.

Team 245
Authors: Slavko Žitnik[1] and Marko Bajec[1]
Affiliation: [1] University of Ljubljana, Slovenia
e-mail: SZ: slavko.zitnik@fri.uni-lj.si; MB: marko.bajec@fri.uni-lj.si

SHORT SYSTEMS DESCRIPTION
Žitnik and Bajec propose a novel modular named entity recognition system that uses multiple linear-chain conditional random fields as the main entity mention extractors, mention merging with deduplication and meta-classification of the candidate extractions. Following the challenge they improved the micro-averaged F score from 78.0 to 83.2 for the CEM and from 79.2 to 84.6 for the CDI subtask.

During the preprocessing their system transforms the input data into the internal representation and enriches the data with additional labelings (i.e., part-of-speech tagging, shallow parsing and lemmatization). After sentence splitting and tokenization they additionally split tokens, which consist of mixed digits and alphanumeric characters, periodic system elements or if they include Greek characters. The extraction component recognizes chemical mentions in text and provides a confidence value for a labeled mention and its sentence. They propose two tokenization types (i.e., token-based and phrase-based) and two different labelings, which result in building four different CRF models. The phrase-based tokenization includes noun phrases identified by the shallow parser as tokens. Furthermore, the models are trained against two labeling schemes. The first is specific to the CEM class, while the second just identifies the existence of a chemical entity. The result of the extraction step are labeled parts of text, identified as chemical mentions by at least one of the models. At the step of merging and redundancy elimination they identify the same extractions by multiple models and prepare data for meta classification in the next step. For the meta classification of extractions they use a support vector machines algorithm to finally decide whether a merged phrase represents a valid chemical mention or not.

Team 199

Authors: Lutz Weber[1] and Matthias Irmer[1]*
Affiliation: [1] OntoChem GmbH, Heinrich-Damerow-Str. 4, D-06120 Halle (Saale), Germany
e-mail: LW: lutz.weber@ontochem.com; MI: matthias.irmer@ontochem.com
*Corresponding author: MI

SHORT SYSTEMS DESCRIPTION

Chemical Named Entity Recognition with OCMiner

OCMiner is a high-performance text processing system for large document collections ranging from short scientific abstracts, full-text articles and patents up to books. Several linguistic options in OCMiner allow adjusting the quality of annotation results which can be specialized and fine-tuned for the recognition of chemical terms.

OCMiner is a modular processing pipeline for unstructured information based on the Apache UIMA framework [1]. Chemical named entity recognition (CNER) is implemented by integrating a number of different toolbox modules into the OCMiner pipeline. Readers process data from a variety of sources, standardizing the input for further analysis. Analysis engines add further data. Consumers provide the final output - in the case of the CHEMDNER challenge in the BioCreative annotation format.
At the core of the system are analysis engines. First of all, there are some preparatory processes such as language detection, sentence splitting, tokenization, document structure recognition. Then, dictionary-based named entity recognition constitutes the most important module for the CHEMDNER task. We use a high performance dictionary look-up technology with support for very large dictionaries (our chemical dictionary has ca. 34 million entries).[2] It implements specific language and dictionary dependent treatment options. For example, it is adaptable to recognize spelling variations, e.g. spaces/hyphens, diacritics, Greek letters (e.g. “α-amino acid” → “alpha-amino acid”), plural forms, apostrophe s. The dictionary can be fine-tuned with the help of domain-specific blacklists, whitelists and graylists. They include stop words, common words and homonymic expressions. In order to get meaningful annotation results, some terms have to be blacklisted in general. Other terms can be identified as chemical names

in a particular context only (e.g. enumerations). These terms are collected in a "graylist" and are only annotated under specific circumstances. Furthermore, there are conditional black- and whitelists where particular context conditions can be specified.
The chemical dictionary is generated from a compound database which is built from various publicly available sources such as PubChem, MeSH, DrugBank, ChEMBL, among others. It contains chemical structures for over 14 million compounds which are stored together with their most common synonyms such as trivial and systematic names, IUPAC names, drug names. The compounds in the database are assigned to chemical compound classes using our chemistry ontology.
The chemistry ontology constitutes the "backbone" of our system's chemical knowledge. It is an ontology of chemical classes which permits an automated high-quality hierarchical classification of chemical compounds according to their structure or their functional properties [3]. The ontology covers the most relevant upper-level chemical compound classes and substituent group classes. All of them contain chemical structure information in form of SMARTS structural patterns [4], which are the basis of a structure based automatic assignment of chemical compounds to these upper-level classes. We are thus able to annotate a chemical term referring to a compound with its ontological parent classes. This allows for an ontological search of compounds in search engines such as OCMiner [5]. Besides the dictionary look-up of database compounds, our system also makes use of name-to-structure conversion tools (OPSIN [6], ChemAxon [7]).
A special module is dedicated to the recognition of chemical formulas. Commonly used types of chemical formulas are, among others, sum formulas (e.g. C_2H_5O) and constituent formulas (e.g. CH_3-CH=CH_2), as well as mixed forms and abbreviations of substituent groups (e.g. "Me") within them. Our system tries to build a chemical structure (e.g. SMILES [4]) from these expressions. If it succeeds, then the expression in question is very likely to be a valid chemical term.
Additional components handle specific scenarios. For instance, we use an abbreviation annotator which finds expansions of acronyms and abbreviated terms. Another module recognizes expressions like "vitamin A and B" as a coordinated entity and annotates "vitamin A" as such and "B" as "vitamin B". A chemistry-specific module tries to recognize whether a given chemical expression refers to a specific compound, a compound class, a substituent group, or classes thereof [8]. This module considers the annotated text, information about the chemical concept it refers to, and the surrounding context. The knowledge of a chemical term type is used for correcting annotation errors. This is especially useful in case of accumulations of various consecutive annotations which are either combined or deleted, depending on the involved chemical term types.

References

[1] Weber L, Böhme T: Methods, Computer Systems, Software and Storage Media for Handling Many Data Elements for Search and Annotation, US Patent Application Publication US8473501; published March 3, 2011.
[2] Apache UIMA [http://uima.apache.org/]
[3] Bobach C, Böhme T, Laube U, Püschel A, Weber L: Automated compound classification using a chemical ontology, Journal of Cheminformatics 2012, 4(1):40.
[4] SMILES/SMARTS [http://www.daylight.com/]
[5] OCMiner [http://www.ocminer.com/]
[6] Lowe D: Extraction of chemical structures and reactions from the literature, PhD thesis. University of Cambridge; 2012 [http://opsin.ch.cam.ac.uk/]

[7] Bonniot de Ruisselet D: Reliably converting names to structures with ChemAxon tools; 2011 [http://www.chemaxon.com/]
[8] Irmer M, Bobach C, Böhme T, Püschel A, Weber L: Using a chemical ontology for detecting and classifying chemical terms mentioned in texts. In Proceedings of Bio-Ontologies; Berlin 2013. [http://www.bio-ontologies.org.uk/]

Team 222

Authors: Saber A Akhondi[1] and Jan A Kors[1]
Affiliation: [1] Department of Medical Informatics, Erasmus University Medical Center, P.O. Box 2040, 3000 CA Rotterdam, The Netherlands
e-mail: SAA: s.ahmadakhondi@erasmusmc.nl; JAK: j.kors@erasmusmc.nl

SHORT SYSTEMS DESCRIPTION

Recognition of chemical entities: combining dictionary-based and grammar-based approaches

We investigated an ensemble approach to extract chemical named entities from text. We focused on extracting non-systematic chemical identifiers using dictionary-based methods, systematic identifiers using grammar-based methods, and database identifiers using a set of manually defined regular expressions. We mainly considered lexical resources that provide chemical structure information. Using an open source indexing system (Peregrine [1]), we assessed the performance of ten different commercial and publicly available lexical resources (ChEBI [2], ChEMBL [3], ChemSpider [4], DrugBank [5], HMDB [6], NPC [7], TTD [8], PubChem [9], Jochem [10], UMLS [11]), in combination with three different chemical compound recognizers (ChemAxon's Document-to-Structure toolkit [12], NextMove's LeadMine [13], and OSCAR 4 [14]). The effect of different stop-word lists, case-sensitivity matching, and use of chunking information (through OpenNLP chunker [15]) was also investigated. The best combination along with a set of regular expressions was used to extract the compounds. Further improvement was made by expanding the dictionary with the gold-standard annotations from the training set that were missed by our system, and adding all false-positives to our stop-word list. Furthermore, we removed the shorter of two overlapping terms. For the CDI subtask we calculated confidence scores for all recognized terms and used these to rank the mentions. Our analyses show that a combination of ChEBI and HMDB as lexical resources, the LeadMine tool for grammar-based recognition, and the regular expressions, outperformed any of the individual systems. The system is able to provide structure information for most of the compounds that are found.

References

[1] Peregrine [https://trac.nbic.nl/data-mining]
[2] de Matos P, Alcantara R, Dekker A, Ennis M, Hastings J, Haug K, Spiteri I, Turner S, Steinbeck C: Chemical Entities of Biological Interest: an update. Nucleic acids research 2010, 38:D249-254.
[3] Gaulton A, Bellis LJ, Bento AP, Chambers J, Davies M, Hersey A, Light Y, McGlinchey S, Michalovich D, Al-Lazikani B, Overington JP: ChEMBL: a large-scale bioactivity database for drug discovery. Nucleic acids research 2012, 40:D1100-1107.
[4] Pence HE, Williams A: ChemSpider: An Online Chemical Information Resource. J Chem Educ 2010, 87:1123-1124.

[5] Knox C, Law V, Jewison T, Liu P, Ly S, Frolkis A, Pon A, Banco K, Mak C, Neveu V, et al: DrugBank 3.0: a comprehensive resource for 'omics' research on drugs. Nucleic acids research 2011, 39:D1035-1041.
[6] Wishart DS, Knox C, Guo AC, Eisner R, Young N, Gautam B, Hau DD, Psychogios N, Dong E, Bouatra S, et al: HMDB: a knowledgebase for the human metabolome. Nucleic acids research 2009, 37:D603-610.
[7] Huang R, Southall N, Wang Y, Yasgar A, Shinn P, Jadhav A, Nguyen DT, Austin CP: The NCGC pharmaceutical collection: a comprehensive resource of clinically approved drugs enabling repurposing and chemical genomics. Science translational medicine 2011, 3:80ps16.
[8] Zhu F, Han B, Kumar P, Liu X, Ma X, Wei X, Huang L, Guo Y, Han L, Zheng C, Chen Y: Update of TTD: Therapeutic Target Database. Nucleic acids research 2010, 38:D787-791.
[9] Bolton E, Wang Y, Thiessen P, S B: PubChem: integrated platform of small molecules and biological activities. Annual reports in computational chemistry 2008, 12th edition, Washington, DC: American Chemical Society.
[10] Hettne KM, Stierum RH, Schuemie MJ, Hendriksen PJ, Schijvenaars BJ, Mulligen EM, Kleinjans J, Kors JA: A dictionary to identify small molecules and drugs in free text. Bioinformatics 2009, 25:2983-2991.
[11] Bodenreider O: The Unified Medical Language System (UMLS): integrating biomedical terminology. Nucleic acids research 2004, 32:D267-270.
[12] ChemAxon- Document to Structure [http://www.chemaxon.com/products/document-to-structure/]
[13] NextMove Software- LeadMine [http://www.nextmovesoftware.com/products/LeadMine.html]
[14] Jessop DM, Adams SE, Willighagen EL, Hawizy L, Murray-Rust P: OSCAR4: a flexible architecture for chemical text-mining. J Cheminf 2011, 3:41.
[15] Apache OpenNLP library [http://opennlp.apache.org/]

Team 259

Authors: Shuo Xu[1] and Xin An[2]
Affiliation: [1] Information Technology Supporting Center,
Institute of Scientific and Technical Information of China,
No. 15 Fuxing Rd., Haidian District, Beijing 100038, P.R. China
[2] School of Economics and Management, Beijing Forestry University,
No. 35 Qinghua East Rd., Haidian District, Beijing 100083, P.R. China
e-mail: SX: xush@istic.ac.cn; XA: anxin@bjfu.edu.cn

SHORT SYSTEMS DESCRIPTION

We participated in the CEM subtask of the CHEMDNER task, and developed a CEM recognition system on the basis of conditional random field (CRF) model and some open-source NLP toolkits. Our system processing pipeline consists of three major components: pre-processing (merge of special characters, sentence detection, tokenization), recognition (CRF-based approach with optimized cost parameter by cross-validation procedure), and post-processing (rule-based approach and format conversion). Compared with our previous system submitted to the BioCreative CHEMDNER task, word representation of each token is added as new features, and the cost parameter is optimized by 10-fold cross-validation procedure.

Team 214
Description not provided

Team 262
Authors: Utpal Kumar Sikdar[1] and Asif Ekbal[1]
Affiliation: [1]Department of Computer Science and Engineering Indian institute of Technology Patna, Bihar, India
e-mail: UKS: utpal.sikdar@iitp.ac.in; AE: asif@iitp.ac.in

SHORT SYSTEMS DESCRIPTION
Domain-independent Model for Chemical Compound and Drug Name Recognition
Our proposed approaches are based on machine learning algorithms namely, Conditional Random Field (CRF) [1] and Support Vector Machine (SVM) [2]. We perform both identification and classification of chemical names in a single step. Most of the features are identified and implemented without using any deep domain dependent resources and/or tools. The feature are, thus, very general in nature and can be applied to solve other similar kinds of problems. The short descriptions of the features are mentioned below:

Context words: Local contextual information within the window of preceding and succeeding three tokens are used as the feature. This is used with the observation that the surrounding words carry effective information to identify chemical names.
Word prefix and suffix: Prefixes and suffixes of fixed-length character sequences are used as the features. Here, prefixes and suffixes of length up to four characters are considered.
Word length: If the length of an entity is larger than a predefined threshold then it is most likely a chemical name. This feature is introduced with the observation that relatively shorter tokens are not the chemical names.
Infrequent word: A feature is defined that fires if the current word appears infrequently in the training data. This is defined based on the observation that frequently occurring tokens are rarely the chemical name.
Word normalization: Word shapes refer to the mapping of each word to their equivalence classes. Each capitalized character of the word is replaced by 'A', small characters are replaced by 'a' and digits are replaced by '0'.
Orthographic features: We define a number of orthographic features depending upon the contents of the word forms. In total, we define 24 features based on the orthographic constructs.
Informative words: Sometimes the tokens or the sequence of tokens that precede and follow the chemical names could be useful for its identification. From the training set, we extract most frequently occurring words that appear within
the context of wi+2 $_{i-2}$ = wi−2 . . . wi+2 of wi. Thus we create two different lists,

one for the informative words that precede the chemical names and the other contains the informative words that follow the chemical names. Thereafter we define two features that fire for the words of these lists.
Chemical prefix and suffix: We extract most frequently occurring prefixes and suffixes of length 2 from the chemical entities present in the training data. Thereafter two binary valued features are defined that fire if only if the current token contains any of these prefixes and suffixes.
Dynamic NE information: This is the output label(s) of the previous to- ken(s). The value of this feature is determined dynamically at run time.
Unknown token feature: This feature is implemented depending upon the word previously seen in training data or not.
The system was trained with the training data and evaluated on the development data. We define five models as below:
1. Model-1: SVM classifier is trained with all the available sentences of the training data.
2. Model-2: Seven classifiers are built by varying the features in SVM and CRF. All these classifiers are then combined together using majority voting.
3. Model-3: CRF classifier is trained only with those sentences that contain at least one chemical name.
4. Model-4: This model is built by training CRF on a new dataset, formed by merging the training and development sets. The sentences that don't contain any chemical name are not considered.
5. Model-5: Same as the fourth model except in this case few heuristics are used to sort out the various issues of tokenization.
Experiments show that the fourth model achieves the highest performance level with recall, precision and F-measure values of 74.58%, 78.28% and 76.39%, respectively. The rest of the models, i.e. first, second, third and fifth show the F-measure values of 73.69%, 75.12%, 74.49% and 74.61%, respectively. A close analysis show that the shallow parsing features such as Part-of-Speech (PoS) and chunk information could be effective to improve the performance further. Results show that sampling training data by removing the sentences that don't contain chemical names is useful.

Acknowledgement: The authors would like to acknowledge the contribution of Dr. Sriparna Saha, Assistant Professor in the Department of Computer Science and Engineering at IIT Patna.

References
[1] Lafferty, J.D., McCallum, A., Pereira, F.C.N.: Conditional Random Fields: Probabilistic Models for Segmenting and Labeling Sequence Data. In: ICML. pp. 282–289 (2001)
[2] Vapnik, V.N.: The nature of statistical learning theory. Springer-Verlag New York, Inc., New York, NY, USA (1995)

Team 263
Authors: Masaharu Yoshioka[1] and Thaer M. Dieb[1]
Affiliation: [1]Graduate School of Information Science and Technology, Hokkaido University. N-14 W-9, Kita-ku, Sapporo 060-0814, JAPAN

e-mail: MY: yoshioka@ist.hokudai.ac.jp; TMD: diebt@kb.ist.hokudai.ac.jp

SHORT SYSTEMS DESCRIPTION
Ensemble approach to extract chemical and drug named entities using different characteristic CNER systems
We present an ensemble approach to aggregate several Chemical Named Entity Recognizer (CNER) tools with different characteristics (rule based and machine learning based) and different annotation criteria using Conditional Random Field (CRF). In addition, we used post text segmentation processing to solve the mismatches between text segmentation and chemical entities and drug names boundaries. We evaluated the system using BioCreative IV, CHEMDNER Task data set. We could achieve a Micro F-score of 88.06 % on this corpus. We confirmed that aggregating CNER tools with different characteristic and different annotation criteria is generally helpful. However, when there is a good system tuned for a certain task, then adding several CNER tools will add only small improvement to the performance. In addition, we have found that text segmentation considerably affect the performance of the system.

Team 207
Authors: Miji Choi1[2] and Karin Verspoor[1,2]
Affiliation: [1] The University of Melbourne Department of Computing and Information Systems
[2] National ICT Australia Victoria Research Laboratory
e-mail: MC: jooc1@student.unimelb.edu.au; KV: karin.verspoor@unimelb.edu.au

SHORT SYSTEMS DESCRIPTION
Description of NEROC: Named Entity Recogniser of Chemicals for CHEMDNER task
Our system has been developed for chemical and drug named entity recognition (NER) based on a machine learning approach, Conditional Random Fields (CRFs). It consists of three basic components: text pre-processing, feature generation, and the CRF component. The provided training and development datasets for the CHEMDNER task are transformed into the required IOB format for training a CRF model with Mallet [1]. CRF models are trained with different combinations of specific feature sets, such as morphological characteristics, information of preceding/following tokens, character level of 2-,3-,4-grams prefix and suffix, and annotation outputs of existing chemical NER systems, ChemSpot and OSCAR4. We introduce a feature that combines the annotated outputs of ChemSpot and OSCAR4; this is a binary feature indicating whether a given token has been identified by either system as being part of a chemical name. This feature has a significant impact on the performance of our solution, producing substantially improved performance compared to the outputs of the individual systems. In the CHEMDNER task, our system achieved F-score 76% and 75% for the chemical document indexing (CDI) and the chemical-entity mention recognition (CEM) tasks respectively.

References

[1] McCallum, A.K. MALLET: A Machine Learning for Language Toolkit. 2002; Available from: http://mallet.cs.umass.edu/.

Team 219
Authors: Madian Khabsa[1] and C. Lee Giles[1,2]
Affiliation: [1]Computer Science and Engineering

[2]Information Sciences and Technology, The Pennsylvania State University, University Park, PA, USA 16802
e-mail: MK: madian@psu.edu; CLG: giles@ist.psu.edu

SHORT SYSTEMS DESCRIPTION

Summary of Ensemble Information Extraction Approach to CHEMDNER

We summarize the system used by the Penn State team in the CHEMDNER task. We have participated into both the Chemical Entity Mention task, and the Chemical Document Indexing task. Our approach utilizes multiple information extraction packages and combines them into a probabilities framework in an ensemble fashion to predict the terms according to predefined probability threshold.

Based on the success of ensemble methods, we propose a new probabilistic approach for ensembling multiple information extraction systems to extract chemical entity mentions in free text. The ensemble method uses the output of OSCAR4 chemical extractor, ChemSpot, and a modified version of ChemxSeer formula and name extractor. The modified version of ChemxSeer is based on a Conditional Random Fields extractor trained on both the training and development datasets. The extractor makes use of different types of features, including syntactic and orthographic features such as the term itself, the character level n-grams of length 2, 3, and 4. In addition we used pattern based features that catches certain characteristics of the term, such as whether the term is a mixed of numbers and letters, inclusion of hyphen, ending with a number, whether a word is all capital letters, small letters, or a mix of both. These features are detected with regular expressions that set the appropriate feature in the CRF model. Other features check for the existence of the term in dictionary of chemical names, and passes the term to a formula parser that decides whether the term can be a formula or not.

After running each extraction system on the corpus, its precision and recall are computed. The results from all the systems are then merged together, with a probability, i.e. confidence score, assigned to each extracted entity. The probabilities are estimated based on the individual extractors that outputted this entity. For example, the probability of an extracted entity being correct, given that it was extracted by OSCAR4 equals to the precision of OSCAR4 extractor. In the case when we merge the output of two extractors, A and B, the probability of an extracted entity being correct, equals the precision of the joint result form these two extractors. That is, we compute the conditional join probability of the two taggers, A and B. These probabilities are estimated by merging the output of these two extractors. For example, the $P(E \mid A \wedge B)$ where E is an entity that was extracted by both A and B, equals to the precision of an extractor C, where C contains all the entities that were extracted by both A and B. Similarly, the probability of an entity E being a correct chemical entity where E was extracted by A and not B equals, $P(E \mid A \wedge \sim B)$. This also can be estimated as the precision of a classifier D, where D contains all the entities that were extracted by A and not by B. This method is extended to all the three extractors, and the probabilities are estimated by merging all the possible combinations. While this approach requires exponential number of estimates given the number of extractors, it is however very simple to compute these estimates. That is because the expensive part is running the individual extractors that take long time. For details please refer to [1].

The advantages of such approach allow specifying the method for which the user is optimizing, whether it is precision or recall by choosing the threshold probability. Above that, it provides a method for merging the output of multiple classifiers in a way that gives more flexibility than majority voting, or extractor stacking [2], where the output of one extractor is used as a feature

for the input of the second extractor. One of the limitations of stacking when used with CRFs is that it makes the second classifier heavily dependent on the previous classifier, therefore assigning lower weights to other important features [3]. Furthermore, it requires retraining the system every time a new extractor is added to the chain.
Using the described approach we were able to obtain 72% F-measure in our best run, and 82.05 recall on new terms in the testing dataset in for our recall optimized run. After the competition ended, we have made significant improvements to our method that increased the F-measure to nearly 82% now. We plan to release this new extraction tool soon.

References
[1] Khabsa, Madian, and C. Lee Giles. "An Ensemble Information Extraction Approach to the BioCreative CHEMDNER Task." BioCreative Challenge Evaluation Workshop vol. 2. 2013.
[2] Florian, Radu. "Named entity recognition as a house of cards: Classifier stacking." proceedings of the 6th conference on Natural language learning-Volume 20. Association for Computational Linguistics, 2002.
[3] Sutton, Charles, and Andrew McCallum. An introduction to conditional random fields for relational learning. Vol. 2. Introduction to statistical relational learning. MIT Press, 2006.

Team 238
Authors: Ravikumar K.E.[1] and Hongfang Liu[1]
Affiliation: [1] Mayo Team Participation, Department of Health Sciences Research, Mayo College of Medicine, Rochester, MN 55901, USA
e-mail: RKE: KomandurElayavilli.Ravikumar@mayo.edu; HL: liu.hongfang@mayo.edu

SHORT SYSTEMS DESCRIPTION
For the CEM task, we extended BioTagger-GM, a system for gene mention detection, and MedTagger, a system for clinical concept mention detection for detecting chemical entities[1,2]. The important component in BioTagger-GM is to incorporate dictionary lookup results into machine learning. The dictionary lookup was based on the three collections of entity names (Chemical, Gene/Protein, Disease) we gathered for BioCreative Task III. We used three machine learning algorithms: Conditional Random Fields (CRFs), Support Vector Machine (SVM), and Logistic Regression (LR). We have also incorporated a post-processing step to correct parenthetical alignment errors and remove false positives appearing in the training data.

For CDI, we applied the following approaches to determine the confidence measure for ranking chemical entities occurring in a document: i) voting based relevancy score, ii) relative frequency relevancy score, and iii) TF-IDF relevancy score.

The systems were trained using the training set and evaluated on the development set. Among all the methods the SVM alone gave the best Macro F-measure (0.82). While combining all the methods gave the best recall (0.91) but it resulted in very poor precision and overall low F-score. Combining SVM, LR and CRF models yielded very high recall (0.89) and lower precision (0.75).

Our experiment demonstrates that combining multiple machine learning methods can result in improvement in the recall but it also compounded the precision errors. The submission runs for CEMs were generated by combining the training data set and the development data set.

However, the performance of our submission runs was not comparable to the performance obtained during the development due to the messed up UTF8 encoding. We have an ongoing project for the CEM task by experimenting advanced feature representations and will report the updated performance metrics in a planned future manuscript.

References
[1] Torii M, Hu Z, Wu CH, Liu H. BioTagger-GM: a gene/protein name recognition system. J Am Med Inform Assoc. Mar-Apr 2009;16(2):247-255.

[2] Torii M, Wagholikar K, Liu H. Using machine learning for concept extraction on clinical documents from multiple data sources. J Am Med Inform Assoc. Sep-Oct 2011;18(5):580-587.

Team 196
Authors: Andre Lamurias[1] and Francisco M. Couto[1]
Affiliation: [1] LaSIGE, Department of Informatics, Faculty of Sciences, University of Lisbon
e-mail: AL:alamurias@lasige.di.fc.ul.pt; FMC: fcouto@di.fc.ul.pt

SHORT SYSTEMS DESCRIPTION
The T196 system: short systems description paragraph
The T196 system aimed at achieving high levels of precision by applying semantic similarity validation techniques to Chemical Entities of Biological Interest (ChEBI) mappings (Grego and Couto, 2013). Our assumption is that the chemical entities mentioned in the same fragment of text should share some semantic relation. Therefore, each recognized term should have at least another term with a semantic similarity higher than a given threshold, otherwise it is considered to be a recognition error. For measuring semantic similarity the T196 system mapped the recognized terms to ChEBI based on FiGO (Couto et al., 2005). The T196 system recognized the terms using a machine learning approach based on Conditional Random Fields using multiple training sets, one for each type of chemical entities.
Acknowledgements
This work was financially supported by FCT through the financial support of the SPNet project (PTDC/EBB-EBI/113824/2009) and through funding of LaSIGE Strategic Project, ref. PEst-OE/EEI/UI0408/2014.
Additional Contributors: Tiago Grego

References
[1] T. Grego and F. Couto, Enhancement of chemical entity identification in text using semantic similarity validation, PLoS ONE, vol. 8, no. 5 (e62984), pp. 1-13, 2013
[2] F. Couto, M. Silva, and P. Coutinho, Finding genomic ontology terms in text using evidence content, BMC Bioinformatics, vol. 6, no. S1(S21), pp. 1-6, 2005

Team 217
Description not provided

Team 265
Authors: Hong-Jie Dai[1] and Richard Tzong-Han Tsai[2]

Affiliation: [1]Graduate Institute of BioMedical Informatics, College of Medical Science and Technology, Taipei Medical University, Taipei, Taiwan, R.O.C.,
[2]Department of Computer Science and Information Engineering, National Central University, Taoyuan, Taiwan, R.O.C.
e-mail: HJD: hjdai@tmu.edu.tw and RTHT: thtsai@csie.ncu.edu.tw

SHORT SYSTEMS DESCRIPTION
CHEMDNER corpus paper: Annotation process, & corpus preparation, dataset and short summary of each system
We adapted the original system [1] developed in the gene mention recognition task for the CHEMDNER task. The chemical compound and drug named entity recognition was formulated as a traditional sequence labeling problem, and the feature sets developed in our previous work [1] and the conditional random fields model were used to examine the effect of the features on the chemical domain. Based on the recent evaluations performed after the CHEMDNER task, we observed that the feature sets used in the gene mention recognition task including the word, orthographical, part-of-speech, word shape, affix and chunk features, could also be useful in the CEM/CDI task and achieved satisfactory F-scores of 73.27%/75.28% using the IOB tag scheme and the Penn treebank tokenization strategy[1]. Furthermore, after adopting a finer tokenization method in which tokens were separated at punctuations like "-", the location between letters and digits, as well as sites where lower case letters are followed by an uppercase letter, the performance is significantly boosted and resulted in an F-score of 80.87% and 82.13% for the CEM and CDI task, respectively.

References
[1] Tsai RT-H, Sung C-L, Dai H-J, Hung H-C, Sung T-Y, Hsu W-L: NERBio: using selected word conjunctions, term normalization, and global patterns to improve biomedical named entity recognition. BMC Bioinformatics 2006, 7(Suppl 5):S11.
[1] http://www.cis.upenn.edu/~treebank/tokenization.html

Team 177
Authors: Tolga Can[1] and Caglar Ata[1]
Affiliation: [1]Department of Computer Engineering, Middle East Technical University, Ankara, Turkey
e-mail: TC: tcan@ceng.metu.edu.tr; CA: e1347145@ceng.metu.edu.tr

SHORT SYSTEMS DESCRIPTION
Ata and Can (Team 177) developed a purely database querying based solution, DBCHEM, for the CHEMDNER task. DBCHEM uses a database with 145 million entries including compound and drug names, their synonyms, and molecular formulas. PubChem Power User Gateway (PUG) system was used to construct the database. Candidate chemical and drug names are identified by querying a sliding window of size 2-4. Exact matches in the database are assigned a score of 1.0 and matches which are identified via a "like" query is assigned a score based on the

specificity of the "like" query. If a token contains all uppercase letters, it is also queried in the molecular formula database. After analysis of the whole article, the words of query windows with no matches are also queried individually. At the end, a post-processing phase is used to merge consecutive chemical names identified in the article abstract. An English dictionary is used as a list of stop words.

Team 191

Authors: Anabel Usié[*1,2]
Affiliation: [1] Departament Ciències Mèdiques Bàsiques, Universitat de Lleida
Av. Rovira roure nº 80, 25298 Lleida. Spain
[2] Departament d'Informàtica i Enginyeria Industrial, Univesitat de Lleida
C/ Jaume II, nº 69, 25001 Lleida. Spain
e-mail: AU: ana.usie.chimenos@gmail.com

SHORT SYSTEMS DESCRIPTION
A tool for the identification of chemical entities (CheNER-BioC)
CheNER tested various hybrid approaches that combine CRFs, dictionary, and regular expression matching. The dictionaries used in CheNER are built by merging the chemical entities from the corpora provided by the BioCreAtIvE organizers, the SCAI corpora, and from http://www.drugs.com/. After tagging by the CRF(s), a post-processing step is done by using regular expression matching to remove False Positives and "action words" that are often appended at the end of chemical mentions. The approach with the highest performance uses a CRF trained to simultaneously identify SYSTEMATIC, TRIVIAL, FAMILY, ABBREVIATION, FORMULA, and IDENTIFIER names. This CRF distinguishes between different classes of chemical names and tags each class in a different way.

Team 182
Description not provided

Team 225

Authors: Isabel Segura-Bedmar[1] and Paloma Martínez[1]
Affiliation: [1]Computer Science Department, Universidad Carlos III de Madrid
e-mail: ISB: isegura@inf.uc3m.es; PM: pmf@inf.uc3m.es

UC3M introduces a rule-based system that consists of a nine-stage pipeline: (1) entity recognition with the ChemSpot tool [1], (2) expanding semantic information from ChEBI [2], using several semantic relations such as is-a, has-role and others, (3) expanding semantic information from MeSH, (4) analysis with the MetaMap tool [3], (5) semantic classification process to determine candidates for a chemical entity mention, (6) filtering pre-selected candidates by using several gazetteers extracted from different sources such as DrugBank and wikipedia, (7) ANNIE PoS tagger [4], to rule out false positives, (8) filtering by lexical-semantic rules developed considering CHEMDNER guidelines and (9) filtering by advanced lexical rules that classify the entities according to POS analysis, affix processing and multi-word processing.

The best run obtains a precision of 0.601 and a recall of 0.538 (F-measure of 0.577). An outcome of this work is the development of non-existing resources for recognizing chemical entities (e.g. gazetteers and a list of biochemical axes), which are available for the research community.

References

[1] Rockäschel, T., Weidlich, M., Leser, U.: Chemspot: a hybrid system for chemical named entity recognition. Bioinformatics 12(28), 1633-1640 (2012)
[2] Degtyarenko, K., De Matos, P., Ennis, M., Hastings, J., Zbinden, M., McNaught, A., Alcántara, R., Darsow, M., Guedj, M., Ashburner, M.: Chebi: a database and ontology for chemical entities of biological interest. Nucleic acids research 36(suppl 1), 344-350 (2008)
[3] Aronson, A.R.: E_ective mapping of biomedical text to the umls metathesaurus: the metamap program. Proceedings of the AMIA Symposium, American Medical Informatics Association, 17-21 (2001)
[4] Cunningham, H., Maynard, D., Bontcheva, K., Tablan, V.: Gate: an architecture for development of robust hlt applications. Proceedings of ACL, 168-175 (2012)
