# Supplementary material for: The CHEMDNER corpus of chemicals and drugs and its annotation principles
Source: J Cheminform. 2015 Jan 19;7(Suppl 1):S2. doi: 10.1186/1758-2946-7-S1-S2 (PMC4331692; doi:10.1186/1758-2946-7-S1-S2)
Supplement: Additional file 2 [file 1758-2946-7-S1-S2-S2.pdf]

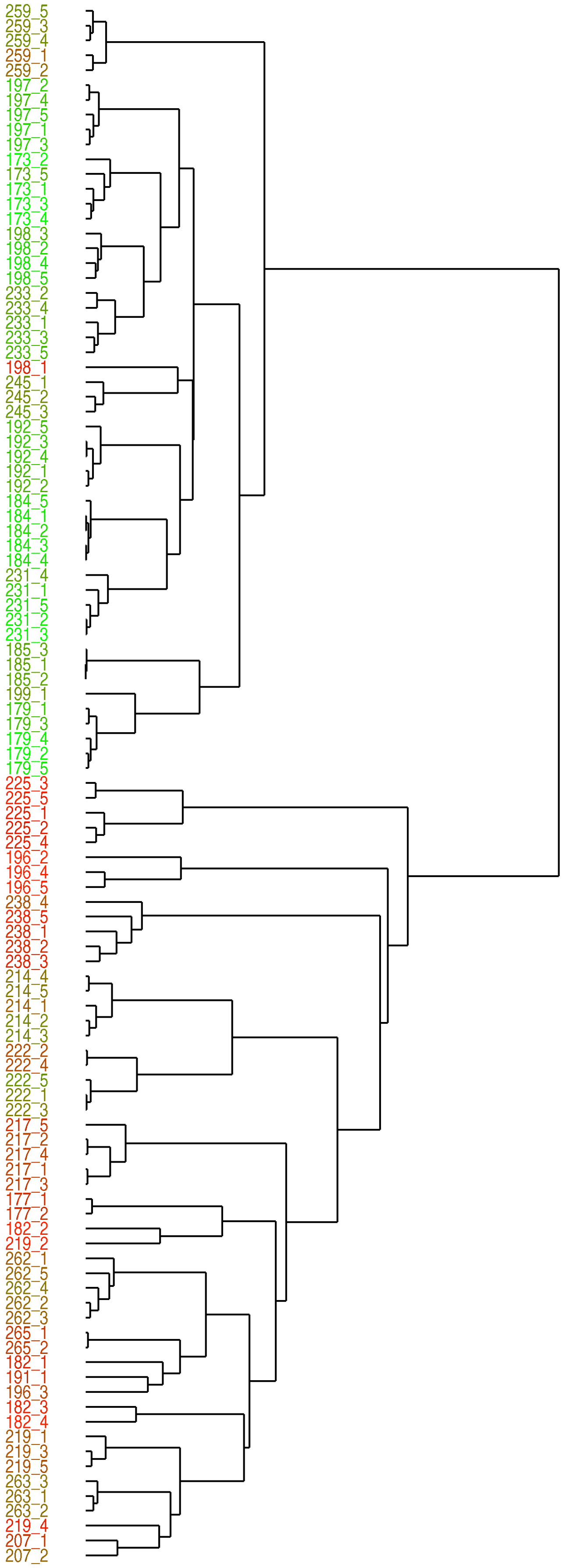

Clustering of team predictions runs for Test set

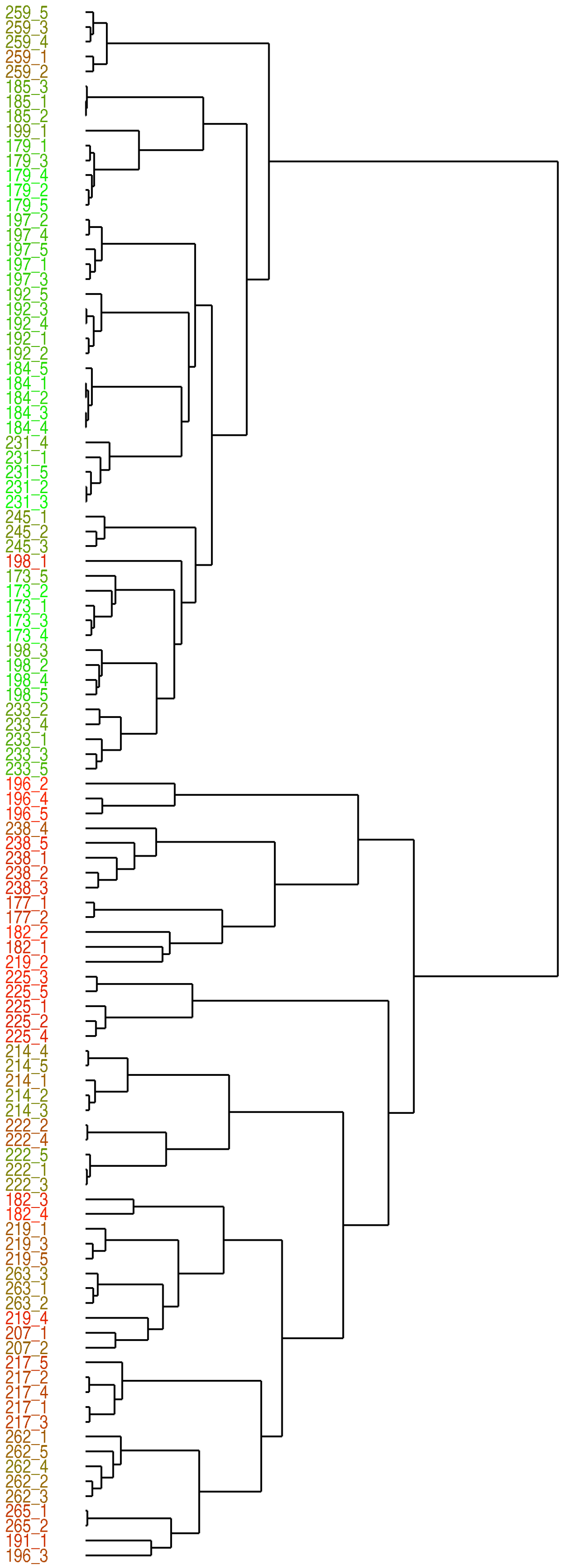

Clustering of team predictions runs for Silver set

Figure X: Clustering of results for the CEM test set. The labels show the team and the run number. Each result is encoded as a vector with as many positions as unique named entities found across all results. Each position contains 1 if the result found that entity or 0 otherwise. The distance is calculated using cosine similarity between the vectors. The F1 scores for each run where used to assign ranks to the results, the rank values where mapped continuously to a gradient from red (worst performing) to green (best performing). The two larger clusters of the dendrogram clearly group runs by performance. Runs from the same team also tend to cluster together.

Figure XI: Clustering of results for the CEM silver set. The same as before test set but using the silver set (see text) predictions. The colors correspond to the performances each run over the test set, that is, exactly as in the previous plot; this assumes that the teams numbered the runs consistently across both sets. Clustering remains similar to the one over the test set.
